# High-throughput phenotyping using hyperspectral indicators supports the genetic dissection of yield in durum wheat grown under heat and drought stress

**DOI:** 10.3389/fpls.2024.1470520

**Published:** 2024-11-22

**Authors:** Rosa Mérida-García, Sergio Gálvez, Ignacio Solís, Fernando Martínez-Moreno, Carlos Camino, Jose Miguel Soriano, Carolina Sansaloni, Karim Ammar, Alison R. Bentley, Victoria Gonzalez-Dugo, Pablo J. Zarco-Tejada, Pilar Hernandez

**Affiliations:** ^1^ Institute for Sustainable Agriculture (IAS), Consejo Superior de Investigaciones Científicas (CSIC), Córdoba, Spain; ^2^ Department of Languages and Computer Science, ETSI Informática, Universidad de Málaga, Andalucía Tech, Málaga, Spain; ^3^ Department of Agronomy, ETSIA (University of Seville), Seville, Spain; ^4^ European Commission (EC), Joint Research Centre (JRC), Ispra, Italy; ^5^ Department of Agricultural and Forest Sciences and Engineering, University of Lleida - AGROTECNIO, Lleida, Spain; ^6^ International Maize and Wheat Improvement Center (CIMMYT), Texcoco, México, Mexico; ^7^ Research School of Biology, Australian National University, Canberra, ACT, Australia; ^8^ School of Agriculture, Food and Ecosystem Sciences (SAFES), Faculty of Science (FoS), and Faculty of Engineering, and Information Technology (IE-FEIT), University of Melbourne, Melbourne, VIC, Australia

**Keywords:** durum wheat, heat, drought, stress, HTP, yield, hyperspectral, GWAS

## Abstract

High-throughput phenotyping (HTP) provides new opportunities for efficiently dissecting the genetic basis of drought-adaptive traits, which is essential in current wheat breeding programs. The combined use of HTP and genome-wide association (GWAS) approaches has been useful in the assessment of complex traits such as yield, under field stress conditions including heat and drought. The aim of this study was to identify molecular markers associated with yield (YLD) in elite durum wheat that could be explained using hyperspectral indices (HSIs) under drought field conditions in Mediterranean environments in Southern Spain. The HSIs were obtained from hyperspectral imagery collected during the pre-anthesis and anthesis crop stages using an airborne platform. A panel of 536 durum wheat lines were genotyped by sequencing (GBS, DArTseq) to determine population structure, revealing a lack of genetic structure in the breeding germplasm. The material was phenotyped for YLD and 19 HSIs for six growing seasons under drought field conditions at two locations in Andalusia, in southern Spain. GWAS analysis identified 740 significant marker-trait associations (MTAs) across all the durum wheat chromosomes, several of which were common for YLD and the HSIs, and can potentially be integrated into breeding programs. Candidate gene (CG) analysis uncovered genes related to important plant processes such as photosynthesis, regulatory biological processes, and plant abiotic stress tolerance. These results are novel in that they combine high-resolution hyperspectral imaging at the field scale with GWAS analysis in wheat. They also support the use of HSIs as useful tools for identifying chromosomal regions related to the heat and drought stress response in wheat, and pave the way for the integration of field HTP in wheat breeding programs.

## Introduction

Wheat is one of the foremost crops around the world, providing around 20% of the global human intake of calories and 20% of protein (FAOSTAT, 2023). It is the most important cereal in Mediterranean agriculture thanks to its adaptation to semi-arid environments, where it is mainly cultivated under rainfed conditions (Arriagada et al., 2020). Moreover, wheat is not only a highly significant crop for its pivotal role in primary production, but also because of the associated food industry chains (Arriagada et al., 2020). These are some of the reasons why there is a rising demand for increased wheat production, linked to the predictions of increasing global wheat requirements (Leegood et al., 2010) and the current geopolitical context (Bentley et al., 2022). However, given the limited availability of land for agricultural use, this increased demand tends to rely mainly on breeding programs focused on breeding crops with higher yield potential and stability under changing environmental conditions (Rufo et al., 2021c). The main constraint on wheat yield mainly originates from stress conditions such as water deficit and high temperature conditions during the grain filling stages, both of which are common in Mediterranean environments (Araus et al., 2002; Barakat et al., 2016). These environments have therefore been identified as a major sensitive region for yield reductions as a result of climate change (Rufo et al., 2021c). Climate change models (IPPC report, 2023) predict a decrease of about 20% in annual precipitations and an increase of approximately 4°C in temperature during the 21^st^ century. Depending on their time and intensity, drought and heat stresses, along with other environmental pressures, can reduce wheat yields from 10% to 90% (Reynolds et al., 2004). For this reason, wheat breeding programs are becoming more focused on the adaptability and stability of productivity in dry areas (Bhatta et al., 2018). The genetic dissection of the complex mechanisms behind the heat and drought response in wheat relies on the availability of suitable phenotyping methods.

Phenotyping using traditional manual methods is currently considered as a bottleneck which prevents faster selection for increased yield and related traits in breeding programs (Araus and Cairns, 2014). This limits our ability to dissect the genetics of critical traits determining yield (Blum, 2011; Cabrera-Bosquet et al., 2012). For this reason, plant breeders need to improve the capacity to phenotype large number of lines rapidly in order to identify superior genotypes accurately (Araus and Cairns, 2014). Breeding populations can include thousands of lines, and accurately assessing and characterizing them simultaneously is a daunting task (McMullen et al., 2009). This is where high-throughput phenotyping (HTP) approaches offer powerful tools to assess phenotypes in large-scale field experiments, using a range of sensors and efficient image-processing systems (Jin et al., 2021; Hussain et al., 2022). HTP integrates equipment for data acquisition, a control terminal and a platform for data analysis, and possesses advantages such as facilitating the non-destructive, high-throughput detection of seen and unseen traits (Berger et al., 2010; Xiao et al., 2022). As a consequence, many plant breeding programs are exploring the use of HTP (Morisse et al., 2022), for example, through the use of vegetation spectral indices, which represent a breeding tool which could improve genetic gains for several plant traits (Babar et al., 2006) or serve as tools for extracting spectral characteristics related to drought-adaptive processes (Condorelli et al., 2018).

Spectral reflectance indices (SRIs) are calculated using reflectance data captured by hyperspectral or multispectral cameras, encompassing the visible (380-740nm) and the invisible near- and short wave-infrared (740-2500nm) regions, and, depending on the spectral domain, these SRIs provide information related to a plant’s photosynthesis and water status (Barakat et al., 2016). Different studies have demonstrated the efficient use of vegetation spectral indices to measure several physiological traits related to crop canopies, such as total dry matter, leaf area index or photosynthetic capacity (Babar et al., 2006; Hassan et al., 2019; Wei et al., 2019), to detect and assess crops under different stress conditions (Aparicio et al., 2002; Condorelli et al., 2018; Camino et al., 2021), or the use of vegetation indices as predictors of crop yield (Sultana et al., 2014; Hassan et al., 2019; Vannoppen et al., 2020) or abiotic stresses (Lowe et al., 2017; Liu et al., 2019) in breeding programs. There is increasing interest in the potential applications of HTP for the genetic dissection of complex traits including yield or drought stress tolerance, through analyses such as QTL mapping or genome-wide association analysis (GWAS). GWAS is a powerful, high-efficiency and high-resolution tool that provides significative associations between molecular markers and traits of interest using empirical models (Xiao et al., 2022).

The combined used of GWAS and HTP at different levels (proximal or remote, in greenhouses or in the field) has great potential for improving our understanding of plant growth and crop breeding (Xiao et al., 2022). Several studies have reported the use of proximal SRIs obtained using handheld devices for GWAS analysis, concluding that they are useful tools to understand the genetic basis of agronomic physiological or quality traits in wheat under yield potential and heat stress conditions both for bread wheat (Gizaw et al., 2016, 2018; Liu et al., 2019; Lozada et al., 2020; Barakat et al., 2021; Krishnappa et al., 2023) and durum wheat (Nigro et al., 2019). HTP based on hyperspectral imaging in greenhouse experiments and GWAS analysis has been recently integrated for dissecting drought traits in bread wheat (Zhang et al., 2024). The use of semi-automated devices in the field increases phenotyping throughput for GWAS, and facilitates the genetic dissection of N deficiency response in bread wheat using sensors with Red-Green Blue (RGB) spectral bands and Near-infrared (NIR) mounted on a tractor (Jiang et al., 2019), and for canopy height and stem elongation rates in winter wheat by using LiDAR (Light Detection and Ranging) on the FIP platform (Field Phenotypig Platform, Kronenberg et al., 2021; Roth et al., 2024). The first report of GWAS analysis using unmanned aerial vehicles (UAVs, UAS, RPAS) was carried out for durum wheat, when the NDVI index was mapped using multispectral imaging (Condorelli et al., 2018). This was followed by the analysis of lodging traits in spring wheat using RGB and multispectral imaging (Singh et al., 2019) and the identification of QTL hotspots for VIs in rainfed wheat (Rufo et al., 2021b).

This study carried out a GWAS analysis using SNP markers (from DArTseq) to identify significant associations for YLD and vegetation spectral indices in elite durum wheat lines grown in Mediterranean environments under drought field conditions. The availability of genome sequences for durum wheat (Maccaferri et al., 2019) and bread wheat (IWGSC, 2018) enabled candidate gene analysis to identify genes involved in key crop processes including photosynthesis, plant stress responses, and hormonal regulation. In this study, we combine, for the first time in wheat, the use of an aerial HTP platform equipped with hyperspectral imaging for field phenotyping, with GWAs analysis of spectral traits, to dissect the genetic basis of yield formation under drought conditions. This approach combines the highest level of spectral resolution (hyperspectral imaging) to derive crop stress indicators with high-throughput capabilities in an aerial platform in the field.

## Materials and methods

### Plant materials and field trials

Field experiments were conducted using a panel of 536 durum wheat genotypes, comprising 494 elite lines from the International Maize and Wheat Improvement Center (CIMMYT) and 42 commercial varieties (**Supplementary Table S1**). The commercial varieties were included as a representative group of wheats adapted to the environmental conditions of the Mediterranean locations assessed in this study. The experiments were grown under rainfed conditions in two locations: Location 1 (37° 32’ 17’’ N, 5° 06’ 57’’ W) (Seville, Spain) in 2014, 2015, 2016, 2017, 2018 and 2021, and Location 2 (37° 27’ 28’’N, 6° 21’ 52’’O) (Huelva, Spain) in 2021. The average maximum and minimum temperatures, together with accumulated rainfall, were obtained from daily data recorded by the agroclimatic stations of the local government, Junta de Andalucía (RIA, 2023), located in the proximities of both locations. The experimental design at each location and for each experiment consisted of an augmented design with two replicated checks for 100 of the elite durum wheat lines, and a three-replicated, randomized, complete block for the 42 durum wheat varieties. For the trials, six individual row plots of 7.2 m^2^ each were used, with a sowing density of 360 seeds/m^2^. The wheat plots were sown between 20^th^ November and 15^th^ December each year and were managed following the standard agricultural practices in both locations.

### DNA isolation and genotyping

The durum wheat lines were sampled at the 4^th^ leaf stage [DC 14 on the Zadoks scale (Zadoks et al., 1974)] for genetic analyses. The plant material was collected at field trials and immediately frozen using dry ice. All the samples were preserved at -80 °C until DNA isolation. About 100mg of the frozen leaf tissue per line was used for DNA extraction with a DNeasy Plant Mini Kit (catalogue number 69104 and 69106) from (Qiagen, Hilden, Germany), following the manufacturer’s protocol. The quality and concentration of each sample was assessed by electrophoresis on a 0.8% agarose gel. In addition, the restriction enzyme Tru1I (Msel, catalogue number ER0982) (ThermoFisher, Waltham, MA, USA) was used to confirm absence of nucleases in DNA prior to genotyping. Approximately 81% of the samples were genotyped by Diversity Arrays Technology Pty Ltd. (University of Canberra, Bruce, Australia) (DArT), and the remaining 19% at the Genetic Analysis Service for Agriculture (SAGA, Mexico). Sequence data for samples genotyped were first aligned against the bread wheat IWGSC RefSeq v2.0 (https://wheat-urgi.versailles.inra.fr/Seq-Repository/Assemblies) and Svevo durum wheat (https://www.interomics.eu/durum-wheat-genome), using end-to-end alignment.

**Figure 1:**
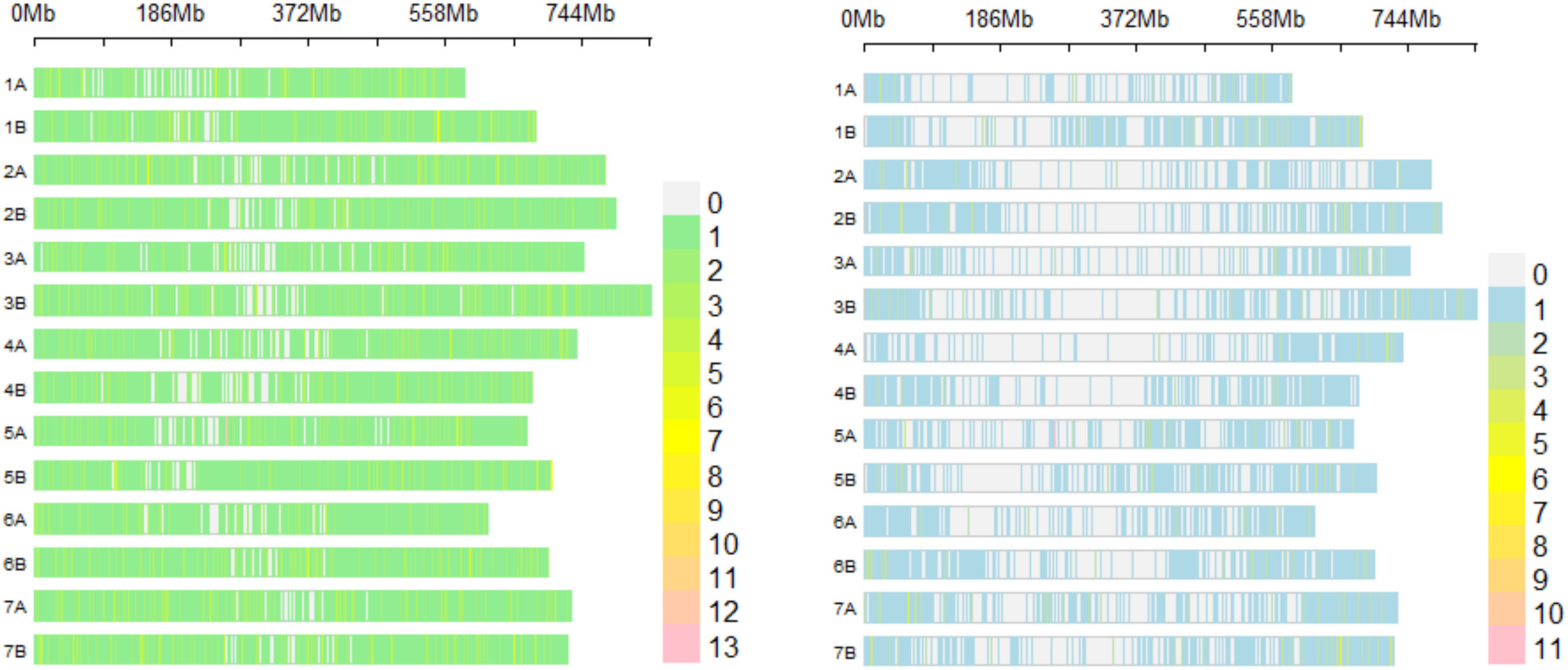
SNP markers density raw dataset (left) and thinned dataset (right) plot chromosome wise representing the number of SNP markers. Horizontal axis shows chromosome length (Mb); color legend depicts number of SNP markers.

A panel of 46,935 biallelic SNP markers was obtained (**Figure 1**). After thinning the marker’s panel by retaining markers with a minor allele frequency (MAF) ≥ 0.05 using Tassel 5 software (Bradbury et al., 2007), the final dataset contained 10,641 biallelic SNP markers (**Figure 1**).

### Phenotypic trait measurement and image acquisition

Yield (YLD; kg/ha) and 19 vegetation spectral indices (**Table 1**) were evaluated across multiple years and environments. We derived the vegetation spectral indices from high-resolution RGB, hyperspectral and thermal remote sensing imagery collected during several airborne campaigns. Hyperspectral imagery was spatially and atmospherically processed to obtain the vegetation indices presented in **Table 1**, following the methods outlined by Zarco-Tejada et al. (2016) and Camino et al. (2019). An aircraft managed by the Laboratory for Research Methods in Quantitative Remote Sensing [QuantaLab, IAS-CSIC, Spain), equipped with a micro-hyperspectral imager (Micro-Hyperspec VNIR model, Headwall Photonics, Fichburg, MA, USA), was used for acquiring the images. The flights were conducted at similar times in the crop cycle (**Supplementary Table S2**) to coincide with the pre-anthesis and anthesis stages of wheat [stages 49 to 69 on the Zadoks scale (Zadoks et al., 1974)]. The spectral vegetation indices used in this analysis (**Table 1**) were classified based on their ability to assess various physiological and structural traits in crop canopies, as follows: 1) Chlorophyll fluorescence indices, which utilize blue (e.g., BF1) and red-edge (e.g., SIF2) spectral regions to monitor photosynthetic capacity; 2) Chlorophyll indices, related to chlorophyll content and essential for assessing photosynthesis (e.g., MCARI), combining visible and near-infrared regions (NIR); 3) Carotenoid indices, reflecting the presence of carotenoids that protect against oxidative stress using green and red-edge spectral regions; 4) Xanthophyll indices, related to light management and photoprotection, such as the Photochemical Reflectance Index (PRI), which primarily utilizes the green spectral region around 550 nm; 5) Plant disease indices, assessing physiological responses to pathogens; and 6) Structural indices, which are related to biomass, leaf area, and overall structural characteristics, focusing on the red and NIR.

**Table 1:** Spectral indices assessed for durum wheat panels grouped by index type (in bold).

Spectral Index	Acronym	Reference
Photosynthetic Activity and Chlorophyll Fluorescence emissions		
Blue fluorescence index	BF1	Zarco-Tejada et al. (2018)
Blue fluorescence index	BF2	Zarco-Tejada et al. (2018)
Solar-induced chlorophyll fluorescence	SIF2	Plascyk and Gabriel (1975); Moya et al. (2004)
Reflectance curvature index	CUR	Zarco-Tejada et al. (2000)
Chlorophyll pigments		
Blue/green index	BGI1	Zarco-Tejada et al. (2005)
Blue/green index	BGI2	Zarco-Tejada et al. (2005)
Carotenoid xanthophyll pigment index	DCabxc	Datt (1998)
Transformed chlorophyll absorption in reflectance index/Optimized soil-adjusted vegetation index (TCARI/OSAVI)	TCARI/OSAVI	Haboudane et al. (2002)
Normalized phaeophytinization index	NPQI	Barnes et al. (1992)
Modified chlorophyll absorption in reflectance	MCARI	Haboudane et al. (2004)
Transformed chlorophyll absorption in reflectance index	TCARI	Haboudane et al. (2002)
Carotenoid pigments		
Simple ratio carotenoids – CARter index	CAR	Hernández-Clemente et al. (2012)
Carotenoid concentration index	CRI700	Gitelson et al. (2003, 2006)
Carotenoid concentration index	CRI700m	Gitelson et al. (2003, 2006)
Carotenoid concentration index	CRI550	Gitelson et al. (2003, 2006)
Carotenoid concentration index	CRI550m	Gitelson et al. (2003, 2006)
Xanthophyll indices		
Photochemical reflectance index	PRI	Gamon et al. (1992)
Carotenoid and Xanthophyll pigments		
Carotenoid xanthophyll pigment index	DCabxc	Datt (1998)
Assessing Plant Health and Disease Stress		
Health index	HI	Mahlein et al. (2013)
Structural and biomass changes		
Normalized difference vegetation index	NDVI	Rouse et al. (1973)

### Population structure and linkage disequilibrium assessment

The thinned molecular markers dataset was used to assess the population structure by principal components analysis (PCA) in Tassel 5.0 (Bradbury et al., 2007). The results were then plotted in R using the ‘*plot’* function (R Core Team, 2020).

Linkage disequilibrium (LD) between pairs of genetic locations across the two wheat sub-genomes (A, B) were evaluated using Tassel 5 (Bradbury et al., 2007). Pairwise LD (square allele frequency, r^2^) for SNP markers pairs was calculated following Weir (1997). The intersection of the fitted curve with the cut-off threshold was the mean r^2^ value for each chromosome (Breseghello and Sorrells, 2006; Liu et al., 2017). LD decay was then plotted in R following Remington et al. (2001) using the mean r^2^ value of each chromosome and the genetic distance in base pairs (bp).

### Statistical analysis and marker-trait associations

Phenotypic correlations between assessed traits were analyzed by the ‘cor’ function in R (Kendall, 1938, 1945; Becker et al., 1988) across years and environments, and also plotted in R using the ‘*ggfortify*’ package (Horikoshi and Tang, 2016).

GWAS was conducted across years and environments using best linear unbiased estimates (BLUEs) for YLD and 19 spectral indices, and 10,641 SNP markers to identify marker-trait associations using the Tassel 5.0 software (Bradbury et al., 2007). A weighted mixed linear model (W-MLM) (Casstevens and Wang, 2015) was applied using the PCA matrix, with the first and second principal components as fixed effects and the kinship matrix (K-mat) (**Supplementary Table S3**) as a random effect, at the optimum compression level and following the model equation:


y=Xβ+Zµ+ϵ

where *y* is a vector of observed phenotypes; *X* and *Z* are matrices related to *β* and *µ*, respectively; *β* is a vector of fixed effects; *µ* is a vector of genetic effects (with covariance proportional to a kindship or relationship matrix); and *ϵ* is a vector of residuals. R was used to extract significant MTAs between markers and assessed traits, according to a Bonferroni-corrected threshold of -log_10_ (0.05/n) = 5.33, where n is the total number of SNPs (10,641), and α = 0.05. Manhattan and quantile-quantile (QQ) plots were visualized using the R package ‘*Cmplot’* (Yin et al., 2021) (script can be found at https://github.com/YinLiLin/CMplot).

### Candidate gene analysis

As described in Mérida-García et al. (2020), the sequences of associated SNP markers were blasted against the bread wheat reference assembly RefSeq v2.0 (https://wheat-urgi.versailles.inra.fr/Seq-Repository/Assemblies) and the Svevo durum wheat reference assembly (https://www.interomics.eu/durum-wheat-genome), with no indels or mismatches allowed, using an *ad hoc* Java program to confirm the physical mapping location on each genome. To estimate the position of the MTAs, measured in centimorgans (cM), a map of correspondences between the positions in bp and cM was created for every Svevo chromosome. This map uses the data provided in Supplementary **Table 2** of Maccaferri et al. (2014), which provides a large set of markers, including their nucleotide sequences, and their estimated cM positions on the correct chromosome. To calculate their positions in bp, a BLAST search into the Durum Interomics pseudomolecules (https://doi.org/10.1038/s41588-019-0381-3) was performed (parameter - ungapped). From the resulting map, the only markers retained were those with a public sequence or available for research purposes, and with a single best hit (maximum bitscore) in the correct chromosome. Finally, the map was sorted by chromosome and cM, and checked to remove those markers whose positions in bp were unsorted. Using the resulting map, and knowing the positions in bp of our markers, their positions in cM were interpolated. To compare with the meta-QTL (MQTL) analysis reported by Soriano et al. (2017), the physical position of the MQTLs was inferred based on the closest DArTseq or SNP marker to the MQTL. A confidence interval of 5kbp to the left and right of the marker was established.

**Table 2:** Physical position (cM) for marker-trait associations based on Maccaferri et al. (2014).

Marker	Chr	Pos (cM)	Traits
SNP229	1A	29.0	CAR, CUR, DCabxc, MCARI, NDVI, PRI, TCARI_OSAVI, TCARI
SNP76228	1A	61.0	CAR, CUR, DCabxc, MCARI, NDVI, PRI, TCARI_OSAVI, TCARI
SNP1275	1A	71.0	BF1, BF2, CAR, CUR, DCabxc, MCARI, NDVI, PRI, TCARI_OSAVI, TCARI
SNP1276	1A	71.0	BF1, BF2, CAR, CUR, DCabxc, MCARI, NDVI, PRI, TCARI_OSAVI, TCARI
SNP1481	1A	85.5	BF1, BF2, BGI1, CAR, CUR, DCabxc, MCARI, NDVI, PRI, SIF2, TCARI_OSAVI, TCARI
SNP77275	1A	111.6	CRI700m
SNP2019	1A	123.2	CAR, CUR, MCARI, NDVI, PRI
SNP2648	1B	12.0	CRI550, CRI550m
SNP2830	1B	33.6	MCARI, NDVI, PRI
SNP26551	1B	37.6	CAR, YLD
SNP3098	1B	46	CUR, MCARI, PRI
SNP3549	1B	47.8	BF1, BF2, CAR, CUR, DCabxc, MCARI, NDVI, PRI, TCARI_OSAVI, TCARI
SNP981	1B	48.8	CAR
SNP3877	1B	65.0	YLD
SNP3937	1B	67.5	CUR, MCARI, TCARI
SNP23059	1B	93.6	CAR, MCARI, TCARI_OSAVI, TCARI
SNP5762	1B	136.2	YLD
SNP32258	1B	156.2	YLD
SNP45417	2A	38.2	BF1, BF2, BGI1, CAR, CUR, DCabxc, MCARI, NDVI, PRI, SIF2, TCARI_OSAVI, TCARI
SNP46534	2A	46.6	BF1, BF2, CAR, CUR, DCabxc, MCARI, NDVI, PRI, SIF2, TCARI_OSAVI, TCARI
SNP6223	2A	52.1	MCARI
SNP33554	2A	91.0	CAR, CUR, DCabxc, MCARI, NDVI, PRI, TCARI
SNP6626	2A	96.9	BF2, CAR, CUR, DCabxc, MCARI, NDVI, PRI, TCARI_OSAVI, TCARI
SNP6675	2A	99.9	CUR, PRI
SNP7069	2A	109.6	BF1, BF2, BGI1, CAR, CUR, DCabxc, MCARI, NDVI, PRI, SIF2, TCARI_OSAVI, TCARI
SNP7835	2A	132.2	MCARI, PRI, TCARI
SNP11390	2A	136.2	CAR, CUR, MCARI, PRI, TCARI
SNP8165	2A	151.2	MCARI
SNP8198	2A	154.6	BF1, BF2, CAR, CUR, DCabxc, MCARI, NDVI, PRI, TCARI_OSAVI, TCARI
SNP8232	2A	154.6	CAR, CUR, MCARI, PRI
SNP13427	2A	163.3	BF1, BF2, CAR, CUR, DCabxc, MCARI, NDVI, PRI, TCARI_OSAVI, TCARI
SNP46141	2A	210.8	CAR, CUR, DCabxc, MCARI, NDVI, PRI, TCARI
SNP46142	2A	210.8	BF1, BF2, CAR, CUR, DCabxc, MCARI, NDVI, PRI, TCARI_OSAVI, TCARI
SNP9483	2B	24.7	BF1, BF2, CAR, CUR, DCabxc, MCARI, NDVI, PRI, TCARI_OSAVI, TCARI
SNP9484	2B	24.7	BF1, BF2, BGI1, CAR, CUR, DCabxc, MCARI, NDVI, PRI, TCARI_OSAVI, TCARI
SNP70996	2B	45.3	MCARI, PRI
SNP9901	2B	55.4	BF1, BF2, CAR, CUR, DCabxc, MCARI, NDVI, PRI, TCARI_OSAVI, TCARI
SNP9976	2B	57.7	CAR, YLD
SNP10568	2B	91.3	HI
SNP10840	2B	95.3	BF2, CAR, CUR, DCabxc, MCARI, NDVI, PRI, TCARI
SNP10841	2B	95.3	CAR, CUR, MCARI, NDVI, PRI
SNP46997	2B	101.6	CAR, CRI550m
SNP11217	2B	115.1	CAR, CUR, DCabxc, MCARI, NDVI, PRI, TCARI_OSAVI, TCARI
SNP13388	2B	137.9	BF1, BF2, BGI1, CAR, CUR, DCabxc, MCARI, NDVI, PRI, SIF2, TCARI_OSAVI, TCARI, YLD
SNP43735	2B	166.6	CAR, CUR, DCabxc, MCARI, NDVI, PRI, TCARI
SNP12651	2B	181.6	BF2, CAR, CUR, DCabxc, MCARI, NDVI, PRI, TCARI_OSAVI, TCARI
SNP46683	3A	7.9	CAR, CUR, MCARI, PRI, TCARI
SNP77245	3A	33.6	BF1, BF2, BGI1, CAR, CUR, DCabxc, MCARI, NDVI, PRI, SIF2, TCARI_OSAVI, TCARI
SNP14668	3A	66.8	MCARI
SNP14760	3A	67	CAR, PRI
SNP15000	3A	80.1	CAR, HI, YLD
SNP38516	3A	81.4	CAR, YLD
SNP15180	3A	90.3	BF1, BF2, CAR, CUR, DCabxc, MCARI, NDVI, PRI, TCARI_OSAVI, TCARI
SNP15291	3A	97.4	BF2, CAR, CUR, DCabxc, MCARI, NDVI, PRI, TCARI_OSAVI, TCARI
SNP15292	3A	97.4	CAR, NDVI, PRI
SNP15681	3A	123.1	BF2, CAR, CUR, DCabxc, MCARI, NDVI, PRI, TCARI_OSAVI, TCARI
SNP15835	3A	136.4	YLD
SNP76391	3B	16.9	BF1, BF2, CAR, CUR, DCabxc, MCARI, NDVI, PRI, TCARI_OSAVI, TCARI
SNP16842	3B	25.4	CAR, CUR, DCabxc, MCARI, NDVI, PRI, TCARI
SNP17449	3B	68.3	CAR
SNP17455	3B	69.1	CUR, DCabxc, MCARI, NDVI, PRI, TCARI
SNP17862	3B	81.2	BF1, BF2, CAR, CUR, DCabxc, MCARI, NDVI, PRI, TCARI_OSAVI, TCARI
SNP18017	3B	88.0	CAR, MCARI, PRI
SNP20210	3B	136.9	BF1, BF2, BGI1, CAR, CUR, DCabxc, MCARI, NDVI, PRI, SIF2, TCARI_OSAVI, TCARI, YLD
SNP76785	3B	136.9	BF1, BF2, CAR, CUR, DCabxc, MCARI, NDVI, PRI, SIF2, TCARI_OSAVI, TCARI
SNP76832	3B	136.9	BF21, BF2, BGI1, CAR, CUR, DCabxc, MCARI, NDVI, PRI, TCARI_OSAVI, TCARI
SNP20617	4A	15.5	YLD
SNP21331	4A	57.3	CAR, MCARI, PRI
SNP21648	4A	65.1	CAR, CUR, DCabxc, MCARI, NDVI, PRI, TCARI_OSAVI, TCARI
SNP21687	4A	69.4	BF1, BF2, BGI1, CAR, CUR, DCabxc, MCARI, NDVI, PRI, SIF2, TCARI_OSAVI, TCARI
SNP21759	4A	79.3	BF1, BF2, CAR, CUR, DCabxc, MCARI, NDVI, PRI, TCARI_OSAVI, TCARI
SNP22313	4A	133.9	BF2, CAR, CUR, DCabxc, MCARI, NDVI, PRI, TCARI
SNP23640	4B	41.6	CAR, CUR, DCabxc, MCARI, NDVI, PRI, TCARI_OSAVI, TCARI
SNP24067	4B	52.9	CAR, CUR, DCabxc, MCARI, NDVI, PRI, TCARI_OSAVI, TCARI
SNP25678	5A	20.6	BF1, BF2, CAR, CUR, DCabxc, MCARI, NDVI, PRI, TCARI_OSAVI, TCARI
SNP25731	5A	26.9	BF1, BF2, BGI1, CAR, CUR, DCabxc, MCARI, NDVI, PRI, SIF2, TCARI_OSAVI, TCARI, YLD
SNP16002	5A	27.4	CAR, CUR, MCARI, NDVI, PRI
SNP26048	5A	48.3	CAR, CUR, DCabxc, MCARI, NDVI, PRI, TCARI_OSAVI, TCARI
SNP47527	5A	48.6	CAR
SNP47528	5A	48.6	CAR
SNP47529	5A	48.6	CAR, PRI
SNP47530	5A	48.6	CAR
SNP47531	5A	48.6	CAR
SNP47532	5A	48.6	CAR
SNP47533	5A	48.6	CAR
SNP47536	5A	48.6	CAR, CRI550m
SNP47537	5A	48.6	CAR
SNP26845	5A	90.3	BF1, BF2, CAR, CUR, DCabxc, MCARI, NDVI, PRI, TCARI_OSAVI, TCARI
SNP28567	5B	6.5	BF2, CAR, CUR, DCabxc, MCARI, NDVI, PRI, TCARI
SNP29706	5B	54.4	CAR
SNP29849	5B	68.5	BF2, CAR, CUR, DCabxc, MCARI, NDVI, PRI, TCARI_OSAVI, TCARI
SNP31932	5B	75.9	PRI
SNP32147	5B	146.1	CAR, NPQI, YLD
SNP27817	5B	148.4	BF1, BF2, BGI1, CAR, CUR, DCabxc, MCARI, NDVI, PRI, SIF2, TCARI_OSAVI, TCARI
SNP30955	5B	150.9	BF2, CAR, CUR, MCARI, NDVI, PRI
SNP32334	6A	0.9	CAR, CUR, MCARI, NDVI, PRI
SNP32837	6A	44.3	BF2, CAR, CUR, DCabxc, MCARI, NDVI, PRI, TCARI_OSAVI, TCARI
SNP32939	6A	49.7	BF1, BF2, BGI1
SNP33144	6A	53.2	MCARI
SNP33615	6A	63.5	CAR, CUR, DCabxc, MCARI, NDVI, PRI, TCARI_OSAVI, TCARI
SNP33665	6A	67.3	BF1, BF2, CAR, CUR, DCabxc, MCARI, NDVI, PRI, TCARI_OSAVI, TCARI
SNP34751	6B	21.6	CAR, NPQI, YLD
SNP34891	6B	27.1	BF1, BF2, BGI1, CAR, CUR, DCabxc, MCARI, NDVI, PRI, TCARI_OSAVI, TCARI
SNP34892	6B	27.1	CAR, CUR, MCARI, PRI, TCARI
SNP47538	6B	31.2	CAR, PRI
SNP47539	6B	31.2	CAR
SNP47540	6B	31.2	CAR
SNP47541	6B	31.2	CAR, PRI
SNP35255	6B	45.7	CAR, MCARI, PRI
SNP13219	6B	52.5	BF2, CAR, CUR, DCabxc, MCARI, NDVI, PRI, TCARI_OSAVI, TCARI
SNP37933	6B	76.3	BF1, BF2, CAR, CUR, DCabxc, MCARI, NDVI, PRI, TCARI_OSAVI, TCARI
SNP37996	6B	86.0	BF1, BF2, BGI1, CAR, CRI550m, CUR, DCabxc, MCARI, NDVI, PRI, SIF2, TCARI_OSAVI, TCARI, YLD
SNP36835	6B	86.2	HI
SNP37315	6B	96.7	BGI2
SNP76145	6B	137.2	CAR, CUR, MCARI, NDVI, PRI, TCARI
SNP73562	7A	8.4	YLD
SNP38478	7A	14.3	YLD
SNP38846	7A	53.4	CAR, CUR, MCARI, NDVI, PRI, TCARI
SNP38848	7A	53.4	BF1, BF2, CAR, CUR, DCabxc, MCARI, NDVI, PRI, TCARI_OSAVI, TCARI
SNP40233	7A	113.6	CAR
SNP40908	7A	147.5	CAR, CRI550m, PRI, YLD
SNP76958	7A	170.7	BF2, CAR, CUR, DCabxc, MCARI, NDVI, PRI, TCARI_OSAVI, TCARI
SNP46389	7A	170.8	BF1, BF2, CAR, CUR, DCabxc, MCARI, NDVI, PRI, TCARI_OSAVI, TCARI
SNP41357	7A	172.9	BF2, CAR, CUR, DCabxc, MCARI, NDVI, PRI, TCARI_OSAVI, TCARI
SNP45972	7B	54.0	HI, YLD
SNP43797	7B	96.1	BF1, BF2, BGI1, CAR, CUR, DCabxc, MCARI, NDVI, PRI, SIF2, TCARI_OSAVI, TCARI
SNP44041	7B	109.3	CAR
SNP21473	7B	195.9	CAR
SNP45528	7B	208.3	CAR, CUR, DCabxc, MCARI, NDVI, PRI, TCARI_OSAVI, TCARI

Candidate genes were identified and manually chosen based on their annotations within a window of ±50kbp. Gene expression analyses were performed using the publicly available transcriptomics analyses under different heat and drought stress conditions previously published for bread wheat (Liu et al., 2015; Ma et al., 2017; Gálvez et al., 2019). These results were drawn as a heatmap using the data retrieved by Wheat Expression (www.wheat-expression.com/) and the R package ‘NMF 0.21.0’ (Gaujoux and Seoighe, 2010). The samples analyzed were: (1) seedling samples grown under controlled conditions included in NCBI SRA ID SRP045409 (control, IS; heat and drought (PEG induced drought) stress for 1 and 6 hours, PEG1 and PEG6, respectively) (Liu et al., 2015); (2) samples grown in a shelter and corresponding to NCBI SRA ID SRP102636 (anther stage irrigated leaf phenotype, AD_C; anther stage drought-stressed leaf phenotype, AD_S; tetrad stage irrigated developing spike phenotype, T_C; and tetrad stage drought-stressed developing spike phenotype, T_S) (Ma et al., 2017); and (3) flag leaf samples from field experiments corresponding to NCBI SRA ID SRP119300 (irrigated, IF; mild stress, MS; and severe stress, SS, flag leaves samples) (Gálvez et al., 2019).

## Results

### Agroclimatic conditions

**Figure 2:**
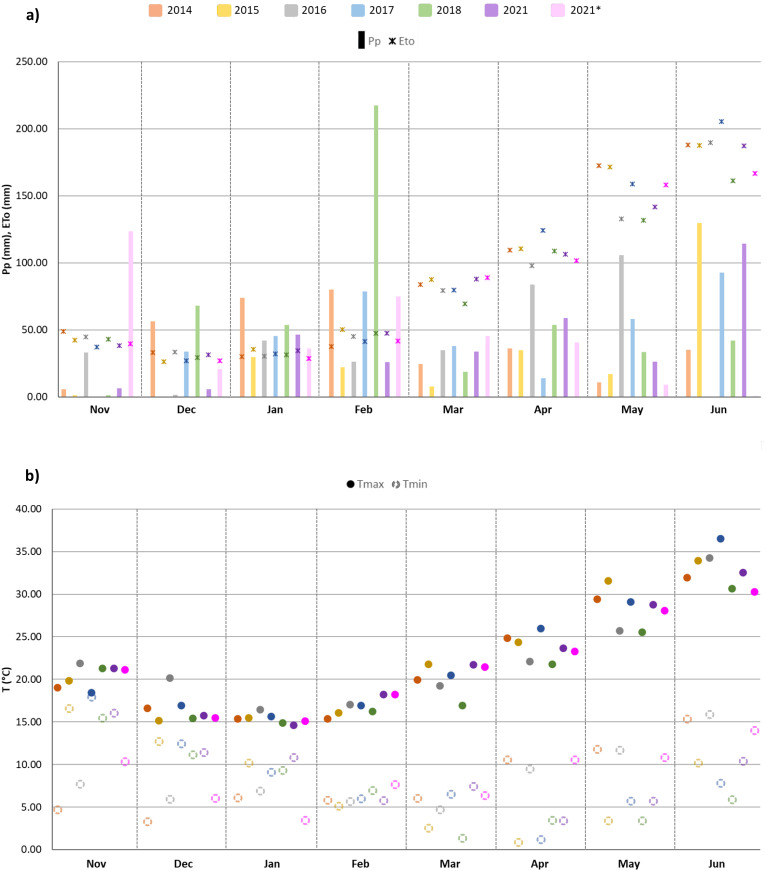
**(A)** Monthly precipitation (Pp, mm) and evapotranspiration (Eto, mm); **(B)** average maximum (Tmax, °C) and minimum (Tmin, °C) temperatures (below) during the crop cycle (from November to June) for each growing season (2014, 2015, 2016, 2017, 2018 and 2021 in Location 1; 2021* in Location 2).

**Figure 3:**
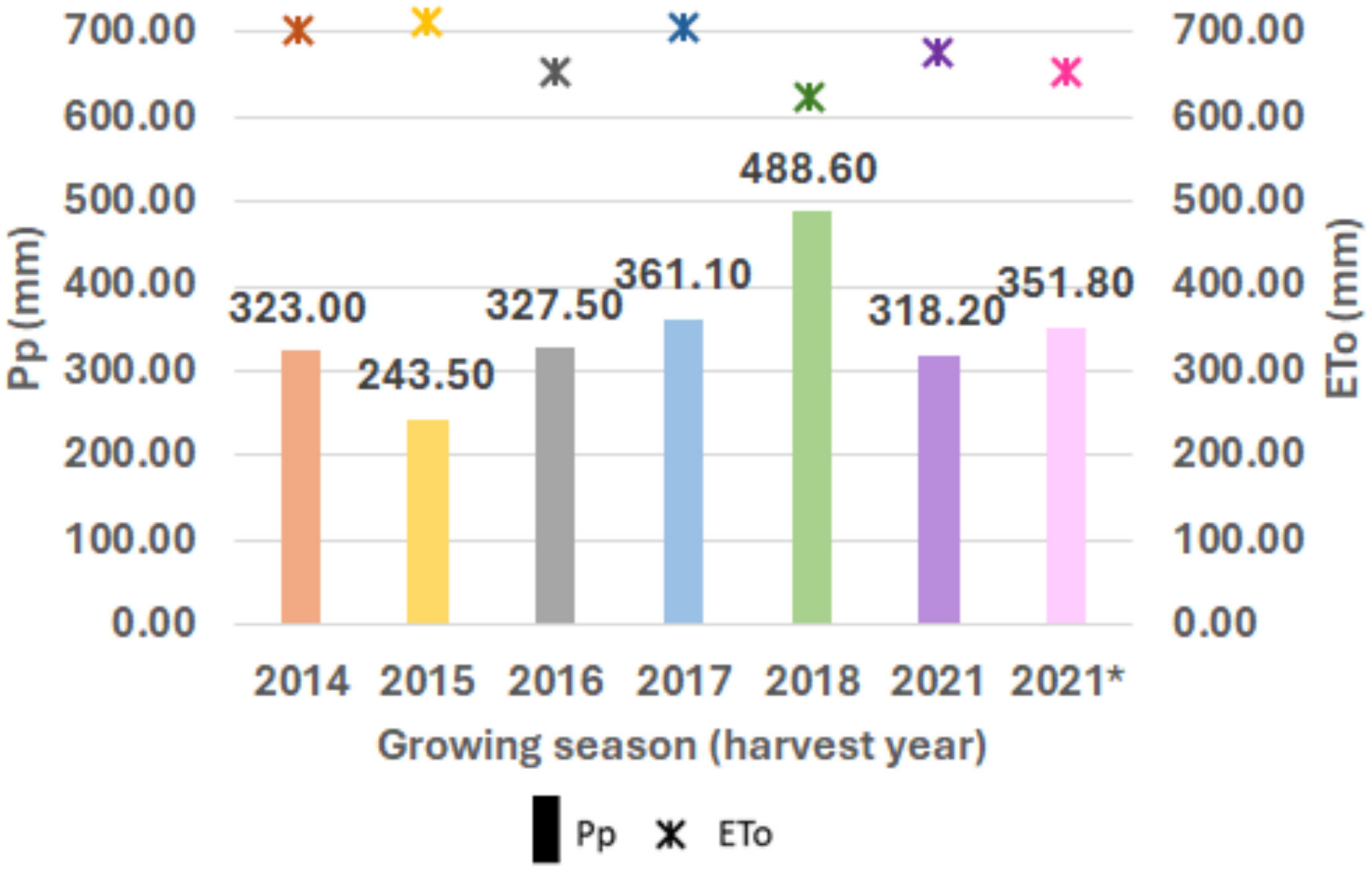
Crop cycle rainfall variation (y-axis) for each growing season (x-axis). Accumulated precipitation (mm) is indicated above each boxplot. Evapotranspiration (mm) at Location 1 for each growing season (2014, 2015, 2016, 2017, 2018 and 2021 in Location 1; 2021* in Location 2).

Locations 1 and 2 are both in Mediterranean climate-zones, characterized by hot and dry summers, and short and mild winters with irregular precipitation. **Figure 2** shows the patterns for maximum and minimum average temperatures (°C) and monthly accumulated precipitation (mm) during the crop cycle (from November to June) for each growing season in the two locations. For Location 1, the 2018 season was the wettest, with 488 mm of precipitation, whereas the driest was 2015, with 243 mm (**Figure 3**).

**Figure 2** reveals increasing temperatures from March until the end of the crop season for all the years assessed, together with irregular precipitation throughout the crop cycle in both testing locations. Yearly variations in precipitation and temperatures were reflected in the differences found in the final YLD (**Figure 3**). However, this relationship was not always clearly evident, with contrasting patterns sometimes being found, as for season 2015 in Location 1 and season 2021 in Location 2 (**Figure 3**), which could be attributed to high soil fertility, as suggested by Royo et al. (2021).

### Phenotypic analyses

**Figure 4:**
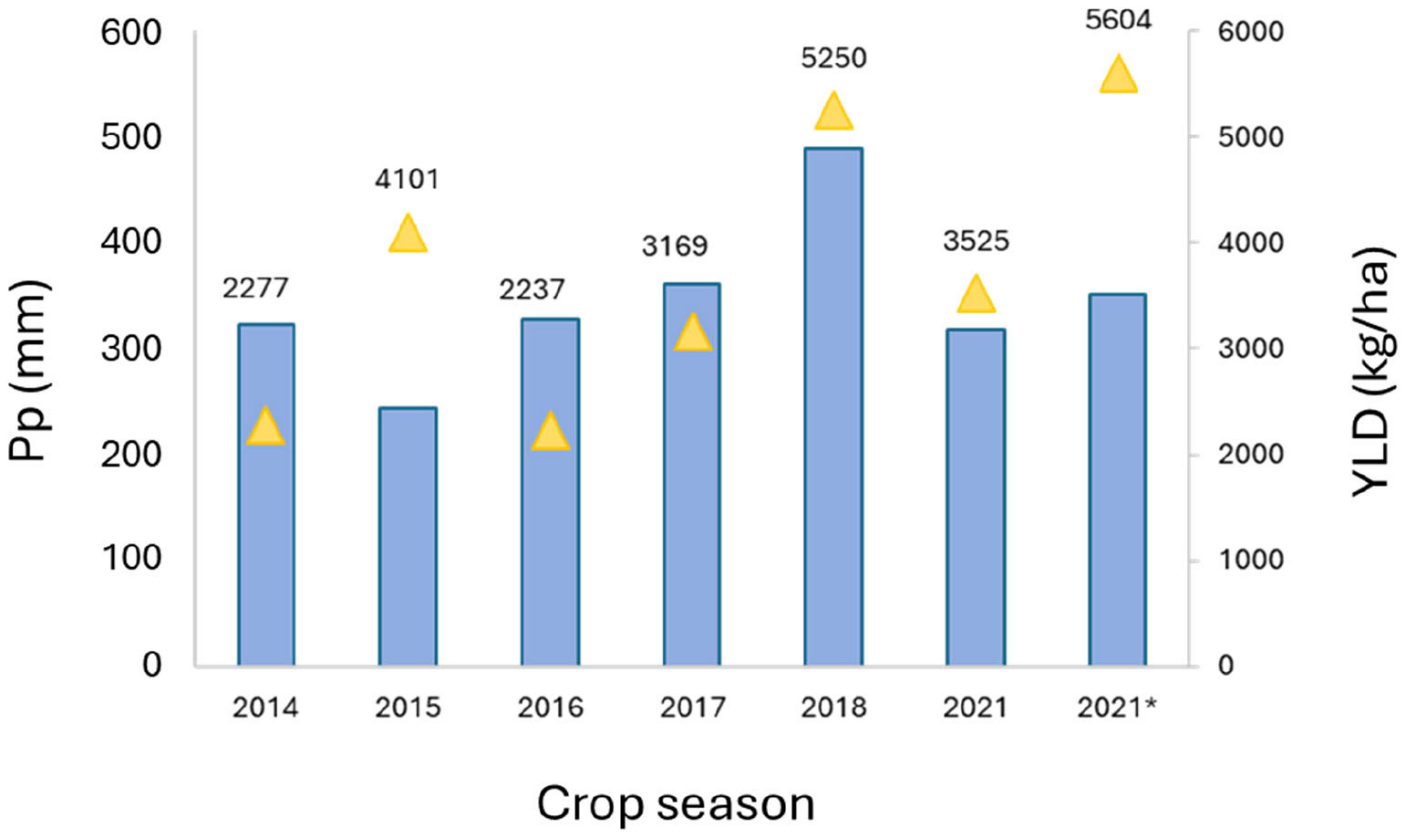
Mean values of yield and crop cycle accumulated precipitation (mm) at Location 1 for each year of assessment. Pp: yearly accumulated precipitation (mm); YLD: yield (kg/ha); 2021*: means corresponded to Location 2 in 2021.

The yearly means of crop final yield (Kg/ha) are shown in **Figure 4**. Variations in precipitation and temperatures were reflected in the differences found in the final yield (**Figure 4**). For instance, Location 1 had the highest values of YLD in 2018 (5,250 kg/ha), likely due to the highest level of accumulated precipitation during the crop cycle (**Figures 3**, **4**). However, this relationship was not obvious in some cases, with different patterns found, as in Location 2 in 2021, with a high average yield (5,605 kg/ha), although the accumulated precipitation (352 mm) was not significantly different from the average yearly rainfall.

**Figure 5:**
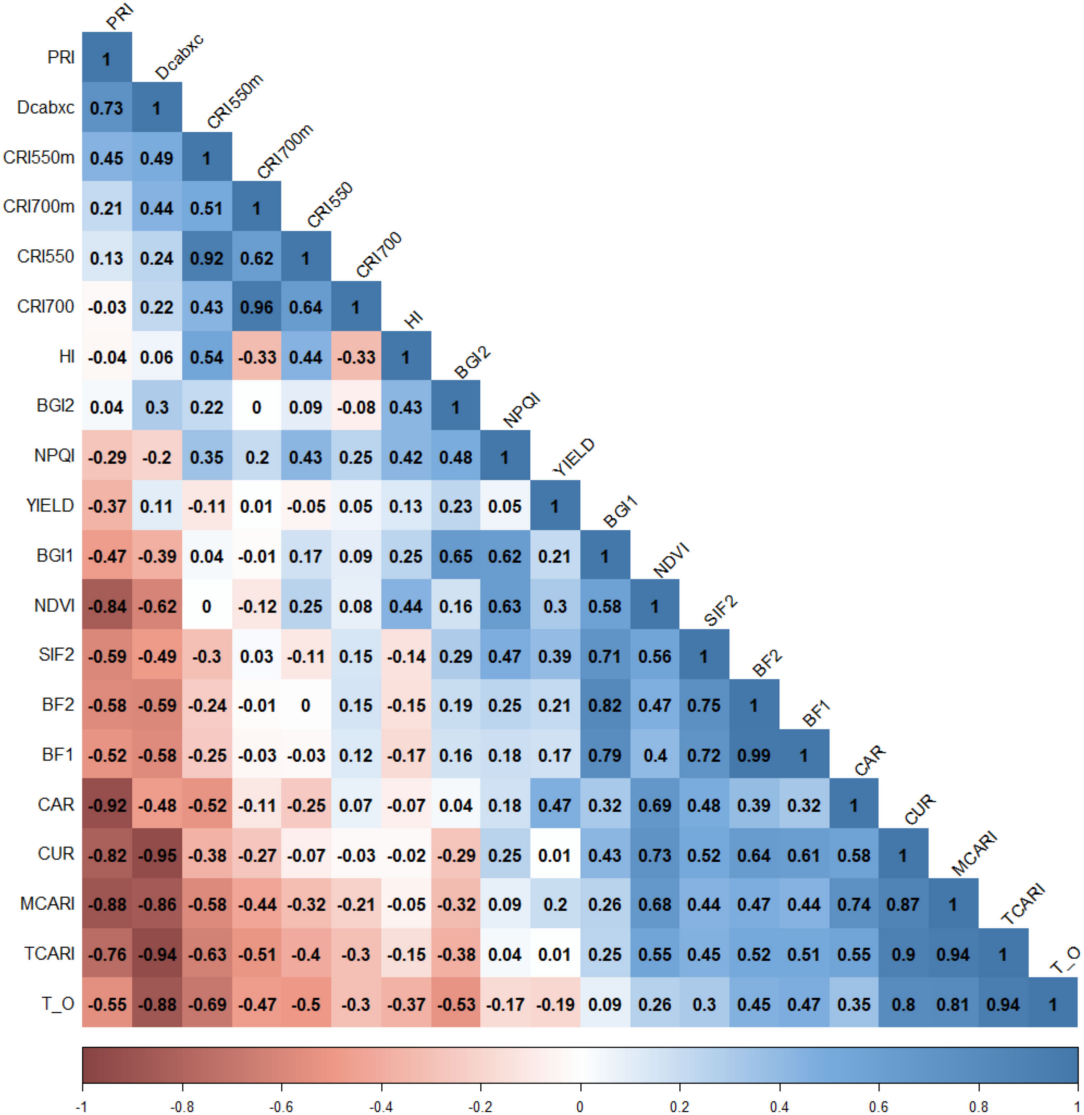
Phenotypic correlations between assessed traits across years and environments. Spectral index abbreviations are shown in **Table 1**. Color intensities show degrees of positive and negative significance (*p*< 0.05).

Phenotypic correlations were found between yield and HSIs related to plant photosynthesis processes, and canopy structure and density (**Figure 5**), which directly or indirectly affected final crop production. Positive correlations (r = 0.30) between YLD and the structural index NDVI (normalized difference vegetation) were also found, as previously reported by Basnyat et al. (2004); Chandel et al. (2019) and Rufo et al. (2021a), using different wheat populations. Moreover, phenotypic correlations were found between YLD and HSIs related to plant photosynthesis processes (indices of simple carotenoid ratio (CAR, r = 0.47), solar-induced chlorophyll fluorescence (SIF2, r = 0.39) and photochemical reflectance (PRI, r = -0.37)) (**Figure 5**). This highlights the important impact of the assimilation processes during grain filling on final yield. As expected, correlations were also found between spectral indices which were classified in the same group (**Figure 5**; **Table 1**).

Significant correlations between HSIs were also observed, several of which were observed between spectral indices belonging to the same categories as described above (**Table 1**). These correlations suggest that these indices capture similar spectral regions that are sensitive to plant traits, such as the chlorophyll region or Solar-Induced Fluorescence (SIF) emission related to photosynthetic capacity. Furthermore, correlations were found between different groups of indices, exemplified by connections between indices of a group of chlorophyll pigments (MCARI, TCARI, and TCARI/OSAVI) with those of a group including photosynthetic activity and chlorophyll fluorescence emission (CUR) (**Figure 5**). Indices from this group were also found to be closely correlated to those of a group of carotenoid and xanthophyll pigments (DCabxc) (**Figure 5**).

### Population structure and linkage disequilibrium

**Figure 6:**
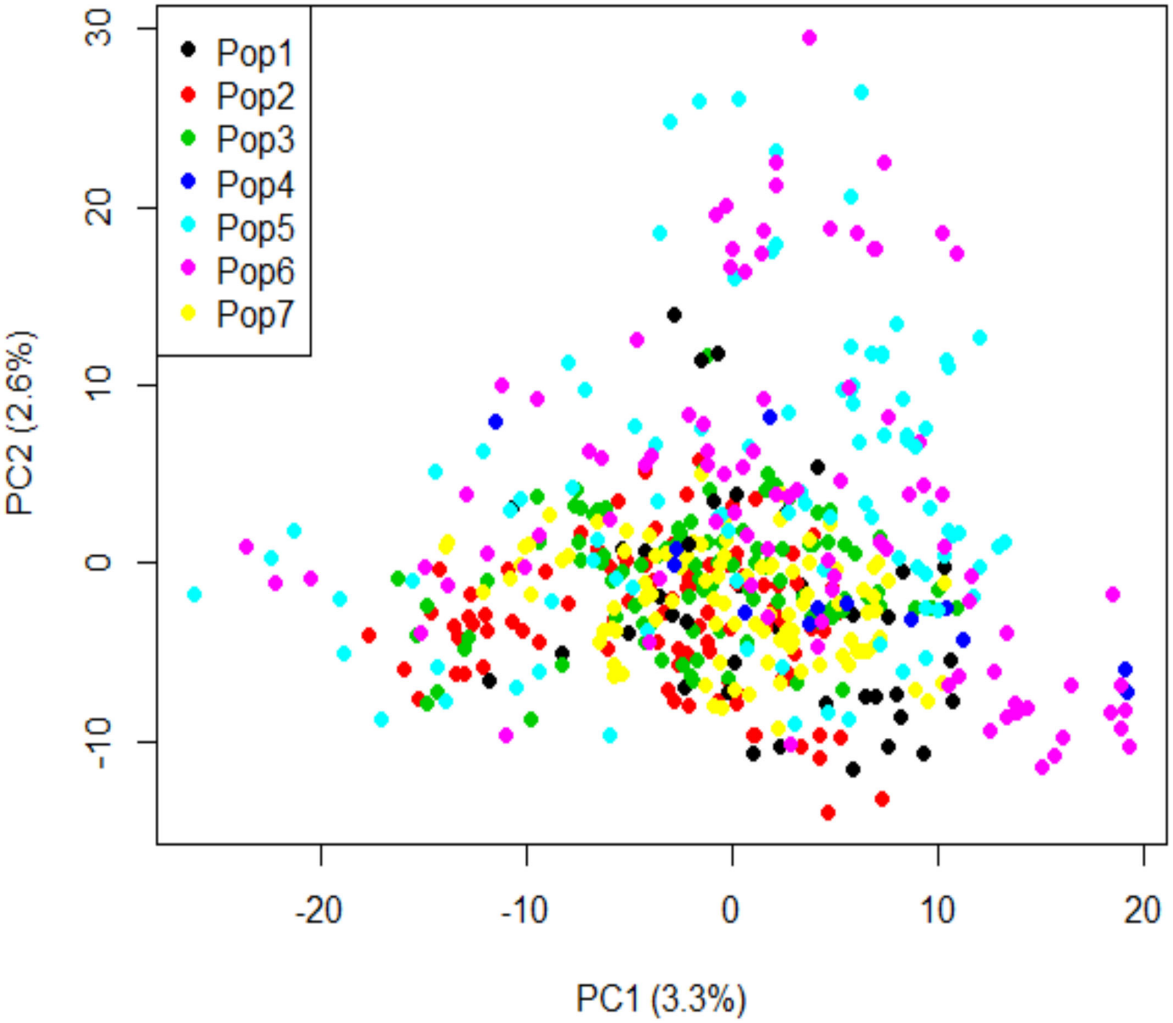
Principal component analysis of genotypic data using 10,641 SNP markers. Pop1: durum wheat varieties; Pop2: elite durum wheat lines collected in 2014; Pop3: elite durum wheat lines collected in 2015; Pop4: elite durum wheat lines collected in 2016; Pop5: elite durum wheat lines collected in 2017; Pop6: elite durum wheat lines collected in 2018; and Pop7: elite durum wheat lines collected in 2021.

**Figure 7:**
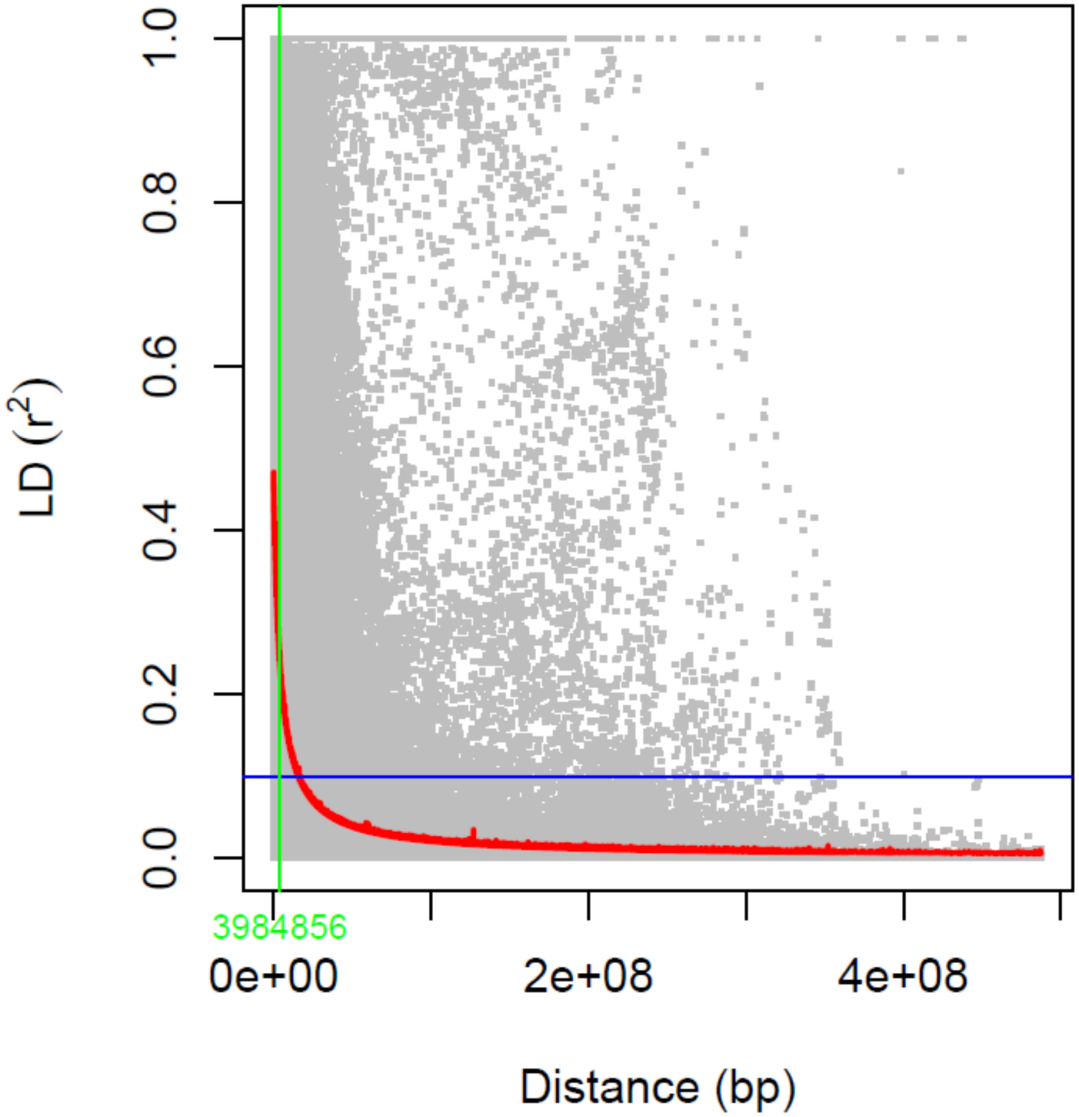
Linkage disequilibrium (LD) decay analysis using SNP markers. Estimated r^2^ against linkage distance in bp is shown. LD decay was measured at 3.98 kbp.

In the PCA analysis, the first and second principal components (PC) accounted for 3.3% and 2.6% of the genetic variation, respectively (**Figure 6**). No genetic sub-structure was identified in the panel. LD decay was estimated around 3.98 kbp for all the chromosomes (**Figure 7**).

### Marker-trait associations and candidate genes

**Figure 8:**
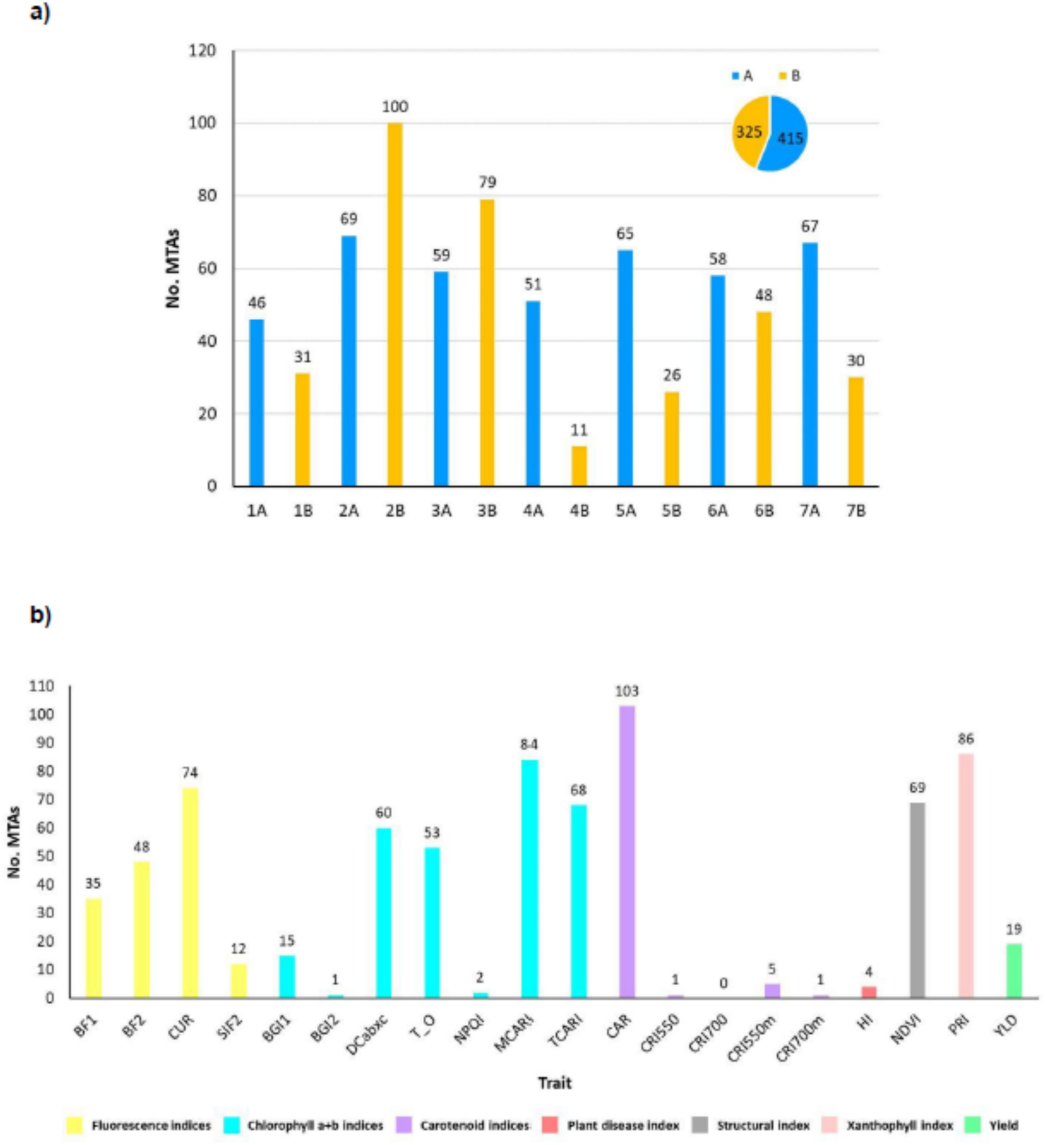
**(A)** Number of MTAs found for each chromosome. Bars for genome A chromosomes are indicated in blue, genome B in yellow; **(B)** Number of MTAs for each trait assessed. Chr: chromosome; No. MTAs: number of significant marker-trait associations; A: durum wheat genome A; B: durum wheat genome B; spectral indices abbreviations are given in **Table 1**; YLD, yield (Kg/ha).

A total of 740 significative marker-trait associations were identified for the 20 analyzed traits (**Supplementary Table S4**). A summary of the results for all the traits across years and environments is reported in **Figure 8**. The physical position of the associated markers is shown in **Supplementary Figure S1**. Manhattan and QQ-plots can be found in **Supplementary Figure S2**. 721 SNP markers were linked to spectral indices (**Supplementary Table S4**) and 19 to YLD. Twelve of the latter were also associated with one or more spectral indices. The carotenoid index (CAR), which was correlated with YLD (**Figure 5**), showed the highest number of significative associations, with 14% of the total number of MTAs, followed by the photochemical reflectance index PRI (11.62%), as well as a moderate correlation with YLD (**Figure 5**), and the chlorophyll index MCARI (11.35%), which is sensitive to chlorophyll a+b content. The indices of CUR (10.00%), sensitive to fluorescence emission, NDVI (9.32%), related to structure, TCARI (9.19%), sensitive to chlorophyll, and DCabxc (8.11%), sensitive to chlorophyll and carotenoids (**Figure 8**), also showed a medium-high number of significative associations (**Figure 8**). Fifteen of the 19 indices analyzed showed co-localization with YLD (**Supplementary Table S7**). Among these, the CAR, PRI, MCARI and CUR indices were those with the highest number of co-localized MTAs, with 32, 25, 23 and 22, respectively. To our knowledge, this is the first report of co-localization of fluorescence (BF1, BF2, CUR, SIF2), chlorophyll a+b (BGI1, DCabxc, TCARI/OSAVI, NPQI, MCARI, TCARI), carotenoid (CAR, CRI550m), plant disease (HI) and xanthophyll (PRI) indices with YLD.

The number of MTAs per chromosome across years and environments ranged from 11 on wheat chromosome 4B to 100 on chromosome 2B (**Figure 8**). Genome A accounted for 54.05% of the total marker-trait associations (320 MTAs), and the remaining 45.95% (272 MTAs) corresponded to genome B.

**Figure 9:**
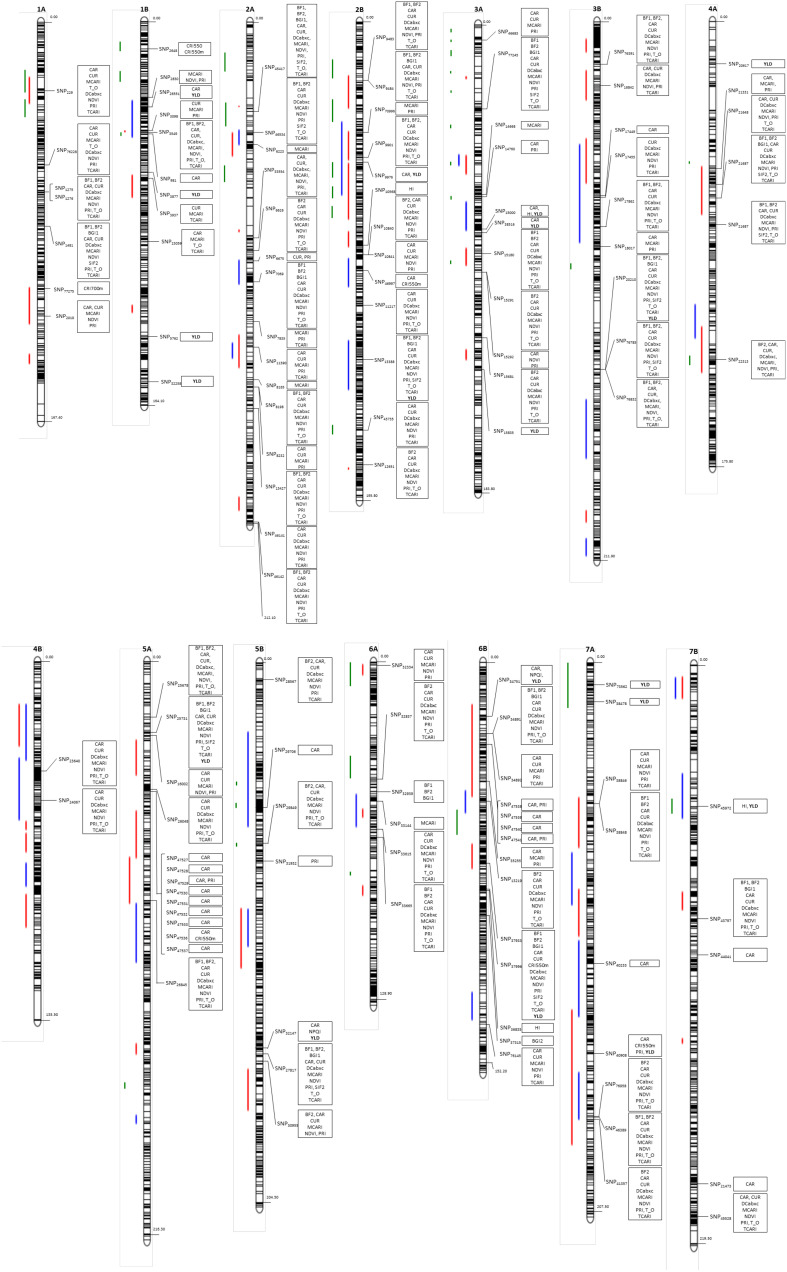
Genetic map for the significant marker-trait associations [position in cM based on Maccaferri et al. (2014)] based on the previously-described yield or yield-related traits (Soriano et al., 2017; Arriagada et al., 2022) for each chromosome. Green: MQTL from Soriano et al., 2017, blue: MQTLs detected under irrigated conditions (Arriagada et al., 2022); red: MQTLs detected under rainfed conditions (Arriagada et al., 2022).

The physical position for MTAs (**Table 2**) and MQTLs, previously described in Soriano et al. (2017) and Arriagada et al. (2022) for each chromosome, are shown in **Figure 9**.

The QTLs found for yield, some of which were shared with one or more vegetation spectral indices, were placed in several chromosomes (**Supplementary Table S4**), which agree with the different QTLs and MQTLs described in previous studies (Soriano et al., 2017; Anuarbek et al., 2020; Arif et al., 2020; Farouk et al., 2021; Mangini et al., 2021; Arriagada et al., 2022; Mulugeta et al., 2023; Valladares García et al., 2023) for durum wheat yield or yield-related traits. Eleven markers (*SNP26551, SNP9976, SNP13388, SNP15000, SNP38516, SNP20210, SNP25731, SNP32147, SNP34751, SNP37996* and *SNP40908*) were found to be significatively associated with yield and the simple carotenoid ratio index (some of them were also associated with other spectral indices, see **Supplementary Table S4**), which were well correlated (r = 0.47) (**Figure 5**), in agreement with the importance and influence of carotenoids on yield as precursors of vitamin-A and plant hormones involved in plant growth and its responses to adverse growth conditions (Mi et al., 2022). Marker *SNP9976*, mapped on durum wheat chromosome 2B, was found within the MQTL15 (Soriano et al., 2017), described for grain weight (GW), and within a grain yield MQTL found under irrigated conditions, as described in Arriagada et al. (2022) (**Figure 9**). *SNP32147* and *SNP34751*, were found to be significantly associated with yield and the normalized phaeophytinization index (**Supplementary Table S4**), which belongs to chlorophyll pigments group (**Table 1**), and is thus related to the process of photosynthesis in the plant. It has been described in previous studies as an efficient SRI for indirect selection of grain yield (Liu et al., 2019). In addition, *SNP32147*, mapped on wheat chromosome 5B, was found in proximity to the MQTL49 (Soriano et al., 2017), related to GY and GW (**Figure 9**). *SNP15000* and *SNP45972* were both significatively associated with yield and health index (**Supplementary Table S4**), which has been previously described as relevant for yield estimation in spring wheat and used to determine patterns of drought distribution in agricultural areas (Zuhro et al., 2020). *SNP15000* and *SNP38516* (also associated with YLD), both mapped on wheat chromosome 3A, were found in the proximity of a YLD MQTL (irrigated conditions) and a yield and yield-related traits MQTL (rainfed conditions), respectively, both described in Arriagada et al. (2022) (**Figure 9**). Finally, *SNP73562* and *SNP38478*, both mapped on wheat chromosome 7A and associated with YLD, were found within the MQTL59 (Soriano et al., 2017) described for GW.

The search for candidate genes aimed to identify corresponding gene models, in durum and bread wheat. We also analyzed the corresponding gene expression under different drought levels, and stress conditions were performed for the significantly associated markers. Gene annotation from the durum wheat genome (https://www.interomics.eu/durum-wheat-genome) allowed the identification of 695 candidate genes (**Supplementary Table S5**). Among these, there were 244 HC genes related to different plant processes including stress responses, but also photosynthesis, and structural and regulatory plant biological processes. Of these, we can highlight the HC genes which encode photosystem I and II assembly proteins, NAD(P)H-quinone oxidoreductases, cytochrome subunits, F-box family proteins, disease resistance proteins, kinase family proteins, aspartic proteinases, or glycosyltransferases, among others (**Supplementary Table S5**). Most orthologs of these genes were also found in gene annotation from the bread wheat reference assembly RefSeq v2.0 (https://wheat-urgi.versailles.inra.fr/Seq-Repository/Assemblies) (**Supplementary Table S6**). The results for the gene expression analyses under different stress conditions (Liu et al., 2015; Ma et al., 2017; Gálvez et al., 2019) are shown as a heatmap in **Supplementary Figure S3**.

## Discussion

This study focused on the phenotypic and yield response of elite durum wheat in field experiments conducted under Mediterranean field conditions. These growing environments are characterized by irregular precipitation during the crop growth cycle, and high temperatures during anthesis and grain filling (Araus et al., 2002; Barakat et al., 2016), exhibiting varying environmental conditions influenced by climate change, specifically characterized by heat and drought.

### Marker trait associations

The dissection of the genetic basis of complex traits is a key objective in breeding programs (Rufo et al., 2021a). In this context, the identification of marker-trait associations as well as QTL related to traits of interest, such as final yield in wheat, are major goals in plant breeding (Arriagada et al., 2020), and can be encompassed within the objectives of these breeding programs. Hyperspectral indices have recently been proposed to assist the genetic dissection of bread (Rasheed et al., 2014; Crain et al., 2018; Chandel et al., 2019; Jiang et al., 2019; Liu et al., 2019; Singh et al., 2019; Rufo et al., 2021a; Govta et al., 2022; Yu et al., 2024; Zhang et al., 2024) and durum wheat traits (Condorelli et al., 2018b; Zendonadi dos Santos et al., 2021; Safdar et al., 2023). The present GWAS analysis resulted in 740 significant MTAs for yield and HSIs, distributed across all durum wheat chromosomes (**Supplementary Table S4**). The pseudomolecule position distribution reveals several QTLs where the HSIs co-locate with yield (**Supplementary Table S7**), highlighting the potential of HSIs as spectral plant traits in yield genomic dissection.

The NDVI (Normalized Difference Vegetation Index), which is an indicator of the plant structure and response to drought (Ji and Peters, 2023) and belongs to the structural and biomass changes indices group, showed 69 associations across almost all durum wheat chromosomes (**Supplementary Table S4**), in agreement with the results presented by Condorelli et al. (2018). Nineteen of them (27.54%) are co-localized with yield associations on several chromosomes (2B, 3A, 3B, 5A, 5B, 6B and 7A). Importantly, *SNP43735*, mapped on wheat chromosome 2B and linked to several HSIs including NDVI, was found within the MQTL19 (Soriano et al., 2017) described for index NDVI. Previous studies, including Xiao et al. (2013) and Li et al. (2014), reported QTL for NDVI which co-locate with YLD on chromosome 3AS. Our results are in agreement with the influence of vegetative growth on final wheat production (Chandel et al., 2019), and thus the relation that can be found between this vegetation spectral index and final crop productivity (Labus et al., 2010). As Li et al. (2020) recently highlighted, water and nitrogen availability can be considered as highly limiting factors in crop production. In fact, nitrogen is the most important element for plant growth and development, affecting the biochemical and physiological functions of the plant and also increasing final yield (Leghari et al., 2016). There are different studies on this topic which used NDVI to predict or estimate final yield in winter and durum wheat, including Chandel et al. (2019), who concluded that the NDVI-YLD relation was stronger in the heading stage (96% accurate estimation of grain and biomass yields in irrigated wheat), and Panek and Gozdowski (2020), whose results agree with Chandel et al., paving the way for forecasting cereal grain yield. *SNP32837* (mapped on chromosome 6A), found in association with several spectral indices including NDVI, was located within the MQTL54 (Soriano et al., 2017) described for GW. Moreover, NDVI has been associated with drought-adaptive traits, as well as grain yield, under stress conditions in wheat crops (Bort et al., 2005; Reynolds et al., 2007; Bowman et al., 2015; Tattaris et al., 2016; Yousfi et al., 2016).

Numerous marker-trait associations were found for HSIs, including indices of group of xanthophyll pigments (86 MTAs), chlorophyll pigments (such as MCARI and TCARI, with 84 and 68 MTAs, respectively) or photosynthetic activity and chlorophyll fluorescence emission (e.g., CUR, with 74 MTAs), among other photosynthesis-related spectral traits (**Supplementary Table S4**). These associations resulted of great interest and value thanks to their relation to photosynthesis processes in plants, as well as to their direct or indirect relation to yield, since, as Paul (2021) highlighted, the role of photosynthesis is pivotal in driving the biological processes involved in final crop yields.

The identification of molecular markers linked to yield and/or different hyperspectral indices facilitates their subsequent use in marker-assisted selection (MAS) or other applications in wheat breeding programs. Moreover, the common associations found for yield and some HSIs can be applied to select vegetation indices as possible estimators of yield, and used for monitoring the development of the crop more efficiently across different growth stages.

The main innovation of this study is the use of high-resolution hyperspectral cameras, opening up new possibilities for exploring a broader spectrum of spectral indices. The versatility of hyperspectral imaging provides researchers with a more comprehensive dataset for characterizing plant traits associated with essential photosynthetic processes. This strategic use of hyperspectral imaging not only advances our understanding of plant physiology but also contributes significantly to the remote sensing community, showcasing its potential to uncover a deeper layer of information for enhanced crop monitoring and phenotypic analysis.

### Candidate gene analysis

This study used hyperspectral indices assessed during the pre- and anthesis stages and final yield phenotyped in durum wheat lines grown under hot, dry, Mediterranean field conditions to perform a combined GWAS analysis. This combined approach unveiled specific genomic regions associated with crop adaptation and yield in response to the challenging climatic conditions of heat and drought.

*SNP1275* (1A chromosome) and *SNP1276* (1B), both significantly linked to various HSIs (**Supplementary Table S4**), were found in the proximity (-8bp) of the durum wheat HC genes *TRITD1Av1G177430.1* and *TRITD1Bv1G163490.1*, respectively. Both genes encode a membrane-associated kinase regulator G, an enzyme which belongs to the protein kinase family, which are involved in plant stress response as regulatory components and in controlled cellular activities (Wang et al., 2020). This agrees with the decreased expression of both genes under increasing drought stress conditions in the field (**Supplementary Figure S3**). Markers *SNP46997* (1B)*, SNP47527, SNP47529, SNP47530, SNP47532* and *SNP47537* (all mapped on chromosome 3B) are all associated with the carotenoid index (CAR), related to the pigment pool involved during the photosynthesis process. These markers were found in the vicinity (within the window of ±50kbp) of several HC genes (**Supplementary Table S6**) which encoded photosystem I P700 chlorophyll a apoproteins and photosystem II CP47 reaction center proteins. The photosystems I and II play important roles in photosynthesis processes (Gao et al., 2018), and carotenoids are part of their co-factors (Fromme et al., 2001; Gao et al., 2018), with some of these genes decreasing their expression under stress conditions (**Supplementary Figure S3**). *SNP3549* (1B), associated with several fluorescence, chlorophyll, structural and xanthophyll indices (**Supplementary Table S4**), was found in the surroundings (within a window of ±15kbp) of gene *TRITD1Bv1G134220.2*, which encodes an ABC family transporter (**Supplementary Table S6**). ABC transporters have been described in plants as proteins which play key roles in plant growth, nutrition, development, response to abiotic stresses, and interaction with its environment (Kang et al., 2011). In keeping with the ABC protein function, this gene decreases its expression under field drought stress conditions (**Supplementary Figure S3**).

The *SNP46534* (2A), associated with different HSIs (**Supplementary Table S4**), was found in the proximity (-3262bp) of the gene *TRITD2Av1G018540.1*, which encodes a cytochrome P450 family protein. It is an enzymatic protein with a key role in plant development and stress defense (Ohkawa et al., 1998; Li et al., 2012; Jun et al., 2015). This gene is also involved in plant development, showing decreased expression under field drought conditions (**Supplementary Figure S3**).

Similarly, *SNP77245* (3A), associated with several HSIs (**Supplementary Table S4**), was found in the surroundings (within the window of ±50kbp) of genes *TRITD3Av1G017220.1, TRITD3Av1G017230.1, TRITD3Av1G017250.1* and *TRITD3Av1G017260.1*, which also encode cytochrome P450 proteins. These genes also showed decreased expression under increased field drought conditions and PEG stress treatment (**Supplementary Figure S3**). A similar reaction in gene expression was found for *TRITD3Bv1G076220.1*, which encodes a cytochrome b559 subunit alpha (**Supplementary Figure S3**), one of the main components of the photosystem II reaction center (Chu and Chiu, 2016). This gene was found in the proximity (-42250bp) of marker *SNP47527* (3B), which is associated with the carotenoid index CAR. In the vicinity (-21,235 bp) of *SNP47529* (3B), associated with CAR and PRI, we found gene *TRITD3Bv1G076150.1*, which encodes a cytochrome b6 (Cytb6), a protein specific to chloroplasts which participates in the electron transport chain in photosynthesis (Cramer, 2020). In the expression heatmap shown in **Supplementary Figure S3**, this gene increased its expression under heat and drought stress field conditions (including IF, control) and under PEG stress treatment, but decreased it under AD_C, T_C and T_S conditions (anther stage irrigated leaf phenotype, tetrad stage irrigated developing spike phenotype and tetrad stage drought-stressed developing spike phenotype, respectively). *SNP46997* (1B), was linked to carotenoid indices CAR and CRI550m. This marker was found in proximity (-17bp) to gene *TRITD1Bv1G206480.4* (**Supplementary Table S6**), encoding a NAD(P)H-quinone oxidoreductase subunit 2, which plays crucial roles in several biological plant processes including photosynthesis (Hu et al., 2018). Moreover, the candidate gene analysis showed another 7 HC genes in the same durum wheat chromosome 1B, albeit more distanced (within a window of -15 and -20kbp) of the *SNP46997* (see **Supplementary Table S6**), which form a cluster (*TRITD1Bv1G206400.1, TRITD1Bv1G206410.1, TRITD1Bv1G206420.1, TRITD1Bv1G206420.2, TRITD1Bv1G206420.3, TRITD1Bv1G206420.4* and *TRITD1Bv1G206430.1*), all of which encode a NAD(P)H-quinone oxidoreductase subunit 1. *SNP5762* (1B), associated with yield, was found in the proximity (-169bp) of gene *TRITD1Bv1G215590.1* (**Supplementary Table S6**), which encodes an aspartic proteinase. This enzyme has been described as part of a group of enzymes related to gliadins in the wheat endosperm (Belozersky et al., 1989). As described in Tenikecier and Genctan (2020), increased or decreased seed size, influenced by endosperm size, affects the final yield in wheat. Moreover, the chromosome where this gene was mapped agrees with previous wheat studies which described major genomic regions for gluten strength and genes related to endosperm proteins as gliadins (Kaan et al., 1993; Ruiz and Carrillo, 1993; IWGSC, 2018; Mérida-García et al., 2019). *SNP9483* and *SNP9484*, both located on wheat chromosome 2B, and *SNP13427* (2A) associated with several HSIs (**Supplementary Table S4**), were found in the proximity (-235 and -772bp, respectively) of HC genes *TRITD2Bv1G013040.1* and *TRITD2Bv1G231900.1* (**Supplementary Table S6**), which encode NBS-LRR (leucine-rich repeats) disease resistance protein, LRRs and immunoglobulin-like domains protein 3 G, respectively. The LRRs are involved as cellular controllers in different plant processes such as cell division or differentiation (Chakraborty et al., 2019), as well as in stress (Torii, 2004; Dufayard et al., 2017) and defense (Lee and Yeom, 2015) responses. A group of 9 markers composed of *SNP33554, SNP8198* and *SNP33554* (2A)*, SNP20210, SNP76785* and *SNP76832* (3B)*, SNP13219* (4A)*, SNP44041* and *SNP73562* (7B) were significantly linked to different HSIs, and some of them also with yield (**Supplementary Table S4**). All were related in terms of greater or lesser proximity (within the window of ±50kbp) to genes encoding F-box proteins (**Supplementary Table S6**), which is one of the largest protein families in plants. F-box proteins can participate as positive regulators in plant responses to stress, such as drought conditions, and also influence plant immunity and hormone signaling (Abd-Hamid et al., 2020).

The marker *SNP15681* (3A), linked to several HSIs (**Supplementary Table S4**), was found in a proximal region (-2662bp) to the HC gene *TRITD3Av1G246000*, which encodes a disease resistance protein responsible for plant immune responses (Belkhadir et al., 2004). *SNP13388* (2B) was associated with different HSIs and yield (**Supplementary Table S4**), and was found in the proximity (-6,765bp) of the HC gene *TRITD2Bv1G222900.1*, which encodes the enzyme glycosyltransferase G. This enzyme is important in plants due to its involvement in photosynthetic processes during the transformation of photosynthesis products into disaccharides, oligosaccharides and polysaccharides (Keegstra and Raikhel, 2001). Moreover, some glycosyltransferases have been described as being involved in the cell wall polysaccharide synthesis of grain wheat endosperm (Suliman et al., 2013). *SNP17449*, (3B), associated with the carotenoid index CAR, was also found in the surroundings (-3470bp) of gene *TRITD7Av1G013810.1*, which also encodes a glycosyltransferase enzyme (**Supplementary Table S6**). None of these genes for glycosyltransferases showed differences in their expression under the different stress conditions assessed (**Supplementary Figure S3**). Finally, *SNP8165* (2A), associated with the MCARI chlorophyll index, was related to gene *TRITD2Av1G258530.1* (-30461bp), which encodes a MYB-related transcription factor, described by Zhao et al. (2018). This enzyme is involved in a plant’s stress responses and increases its expression under PEG6 stress treatment, also showing a slight increase in its expression under T_C and T_S conditions (tetrad stage irrigated developing spike phenotype and tetrad stage drought-stressed developing spike phenotype, respectively) (**Supplementary Figure S3**).

Among the candidate HC genes results obtained using the bread wheat reference genome, the genes found within a ±50kbp window of three SNP markers (**Supplementary Table S4**) were of special interest. *SNP2648* (2D), mapped in durum wheat chromosome 2A and associated with the carotenoid indices CRI550 and CRI550m (both carotenoid indices), was found in the proximity of the HC genes *TraesCS2D03G0083900.1* and *TraesCS2D03G0084000.1*, both of which encode a flower-promoting factor 1-like protein 1. This protein regulates plant flowering, and is also involved in the gibberellin signaling pathway (Kania et al., 1997). The flowering locus has also been previously associated with seed dormancy processes (Chen et al., 2014; Chen and Penfield, 2018), germination (Chiang et al., 2009) and water use efficiency (McKay et al., 2003; Mohammadin et al., 2017), among other plant processes. The orthologs in durum wheat for these genes were *TRITD2Av1G010590* and *TRITD2Bv1G013770*, both of which are located in durum wheat chromosome 2A and encode flowering-promoting factor 1-like proteins 1. Marker *SNP28567* (5B), linked to several HSIs related to photosynthetic processes (**Supplementary Table S4**), was found in the proximity (-5343 and -1454bp, respectively) of two HC genes, *TraesCS5B03G0023300.1* and *TraesCS5B03G0023400.1*, both of which encode an ERD (Early-responsive to dehydration stress) family protein. These ERD genes have been described as those with a rapid activation during drought stress conditions (Alves et al., 2011). The expression of the first gene slightly decreases with increasing stress levels under both field and PEG conditions. However, interestingly, this gene exhibits higher expression levels under PEG treatment compared to stress conditions in the field. *TraesCS5B03G0023400.1*, slightly increases its expression with increased PEG treatment (**Supplementary Figure S4**). The orthologs in durum wheat were *TRITD5Bv1G003930* and *TRITD5Av1G004810* (mapped on 5B and 5A, respectively), both of which encode an ERD family protein. *SNP34891* and *SNP34892* (both mapped on 6B), associated with several HSIs (**Supplementary Table S4**), were found in proximity to 6 HC genes (*TraesCS6B03G0102400.1, TraesCS6B03G0102500.1, TraesCS6B03G0102700.1, TraesCS6B03G0102800.1, TraesCS6B03G0103000.1* and *TraesCS6B03G0103100.1*) (**Supplementary Table S6**), all of which encode high affinity nitrate transporters, which, as their name suggests, play a key role in nitrate uptake (Crawford and Glass, 1998), as well as in nitrate transport and use, and stress resistance (Du et al., 2022). The ortholog genes in durum wheat were *TRITD6Av1G006050*, *TRITD6Av1G006030*, *TRITD6Bv1G008700* and *TRITD6Av1G006000* (mapped on 6A and 6B), all of which encode high affinity nitrate transporters.

## Conclusions

The use of hyperspectral imagery as a high-throughput phenotypic tool to obtain vegetation indices, and their co-localization with final crop yield in GWAS analysis, opens up the possibility of using the HSIs to complement or replace certain field measurements in breeding programs, and of their use as estimators of final production. The GWAS results reported here showed marker-trait associations for final crop yield and HSIs related to photosynthesis processes and structural properties. These results contribute to a better understanding of the dissection of the HSIs assessed, which is directly or indirectly related to final yield or critical physiological processes in durum wheat. Candidate genes analysis revealed a number of gene models across all durum wheat chromosomes, among which we can highlight those related to photosynthetic processes and plant stress responses. The MTAs and candidate genes reported in this study could be of use in breeding programs focused on the use of HTP for driving yield improvements by selecting suitable genotypes. These results support the use of hyperspectral remote sensing imagery in the context of wheat breeding. Further research is needed to advance in our understanding of biophysical modelling to develop spectral plant traits specific to heat and drought resilience.

## Data Availability

The original contributions presented in the study are included in the article/**Supplementary Material**. Further inquiries can be directed to the corresponding author.

## Supplementary material

Supplementary Figure S1 Physical position of the (57) associated SNP markers found in GWAS analysis.

Supplementary Figure S2 Manhattan and quantile-quantile plots for the 19 traits assessed in GWAS analysis.

Supplementary Figure S3 Heatmap for gene expression analysis under several stress conditions for candidate genes. IF: irrigated field conditions; MS: mild stress conditions; SS: severe stress conditions (Gálvez et al., 2019 ***); IS: seedling PEG shock control; PEG1: seedling 1 h PEG stress; PEG6: seedling 6 h PEG stress (Liu et al., 2015 ****); AD_S: anther stage irrigated shelter phenotype; AD_S: anther stage drought stressed shelter phenotype; T_C: tetra stage irrigated shelter phenotype; and T_S: tetrad stage drought shelter phenotype (Ma et al., 2017 *****).
