# Supplementary figures and images for: High-throughput phenotyping using hyperspectral indicators supports the genetic dissection of yield in durum wheat grown under heat and drought stress
Source: Front Plant Sci. 2024 Nov 22;15:1470520. doi: 10.3389/fpls.2024.1470520 (PMC11620856; doi:10.3389/fpls.2024.1470520)

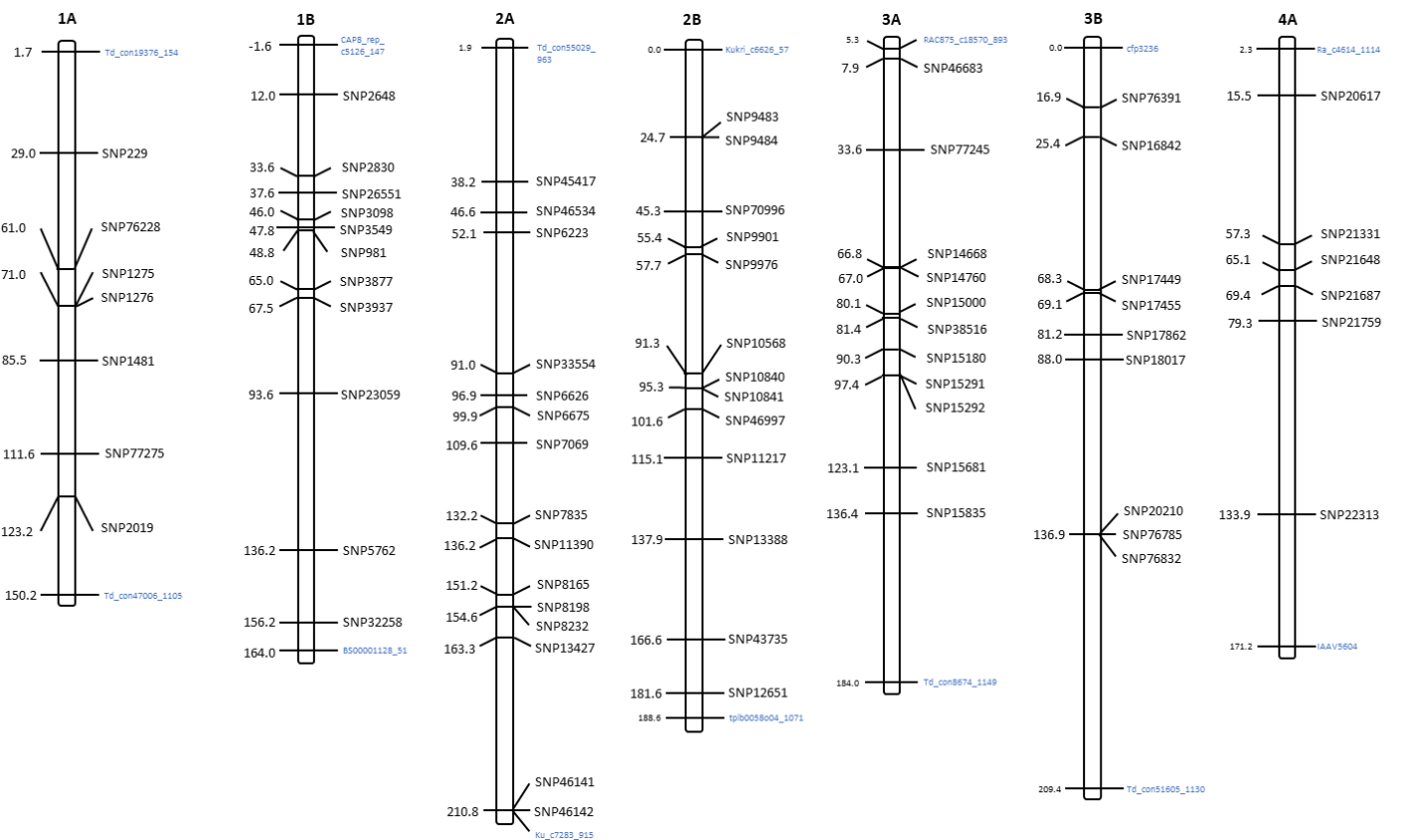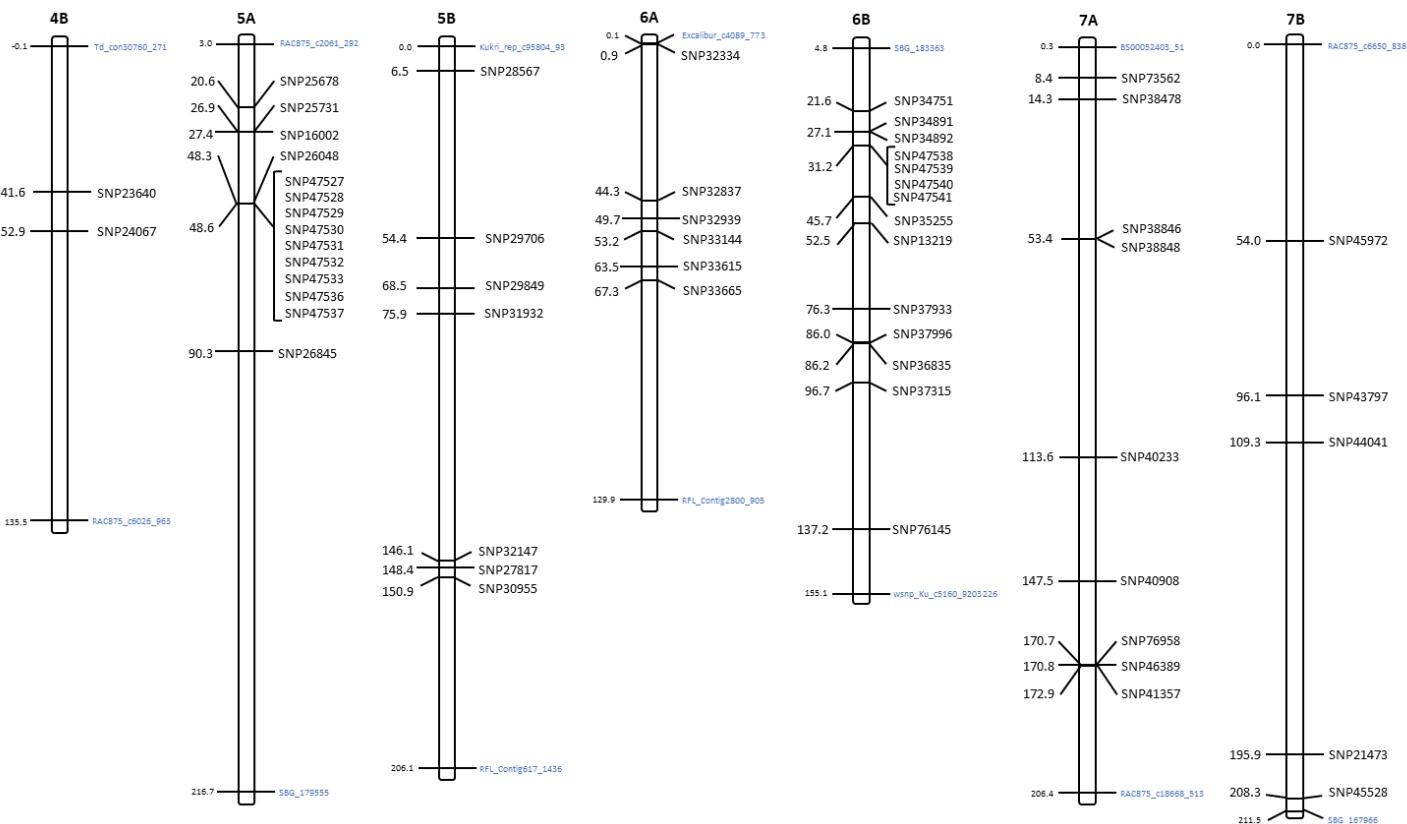

Supplement: Supplementary Figure S1 — Physical position of the (57) associated SNP markers found in GWAS analysis. [file DataSheet1.pdf]

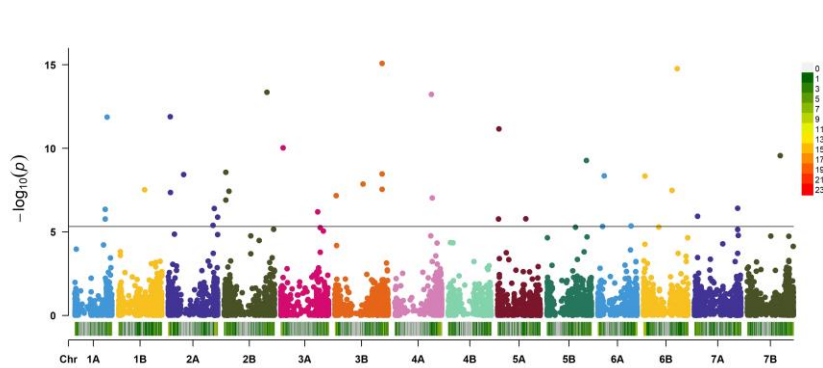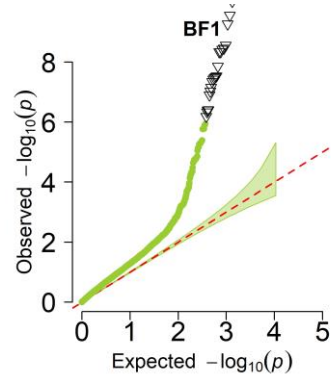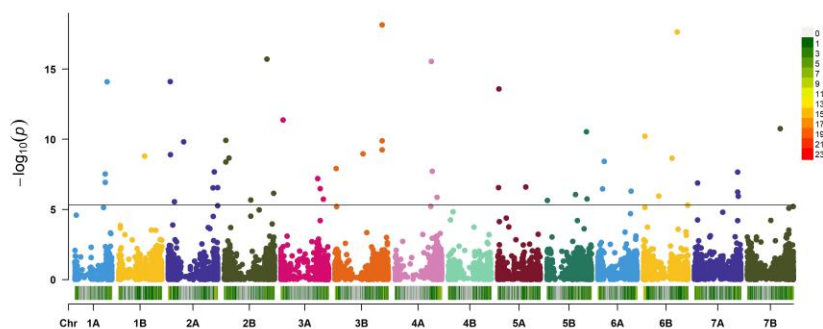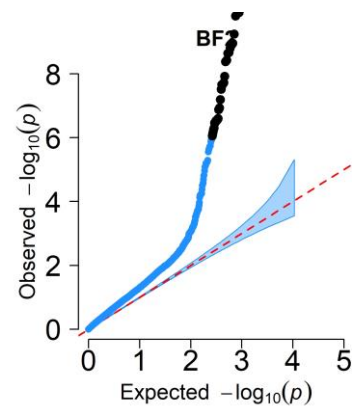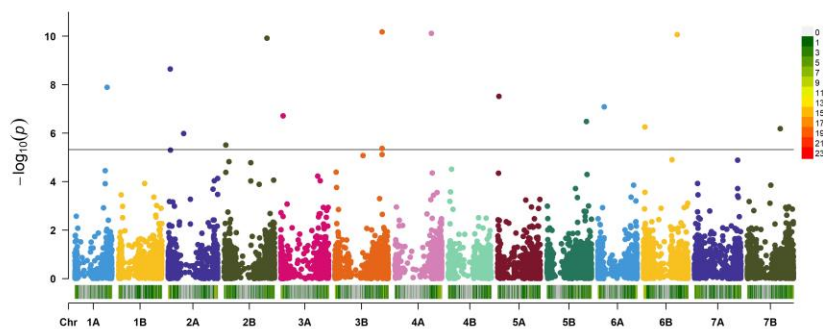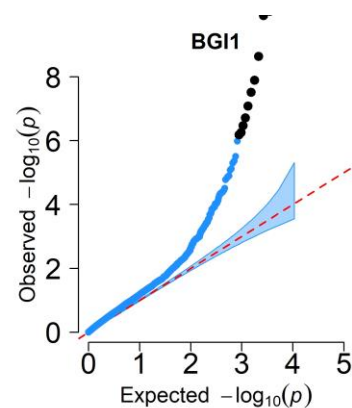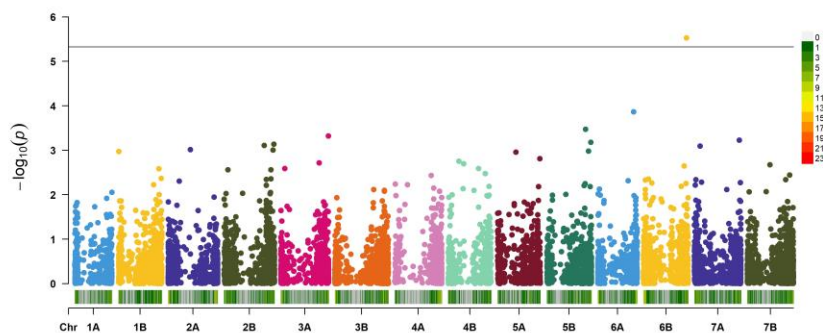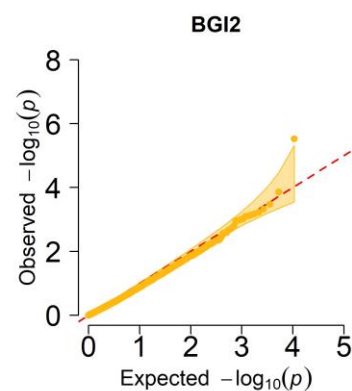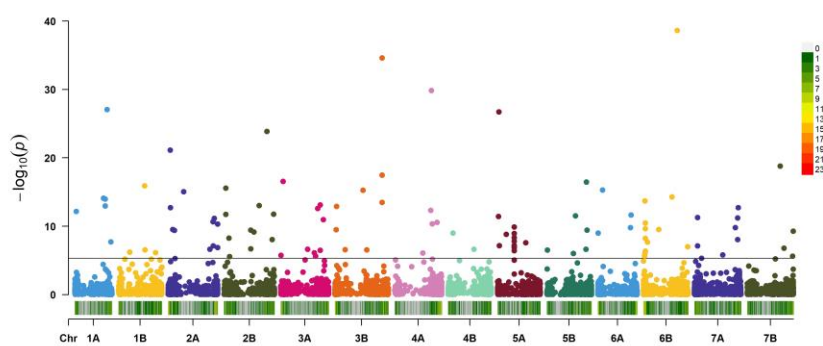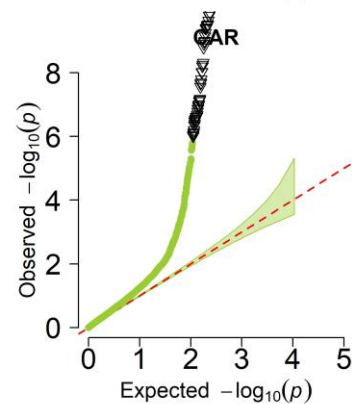

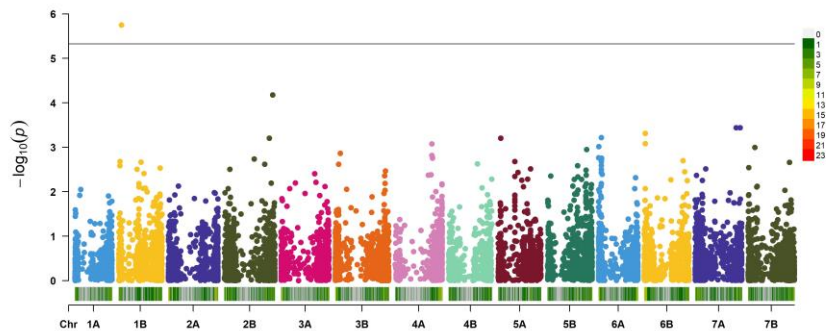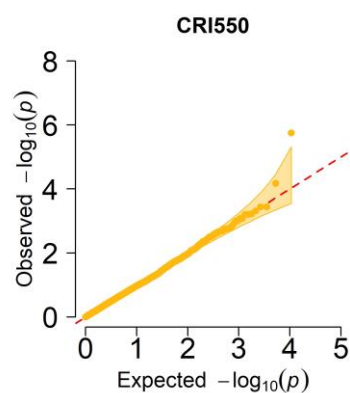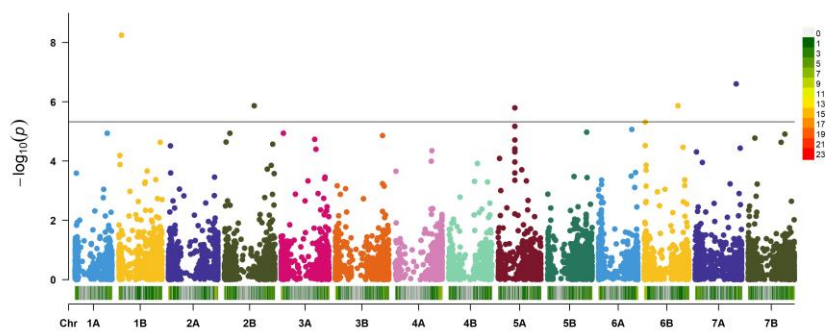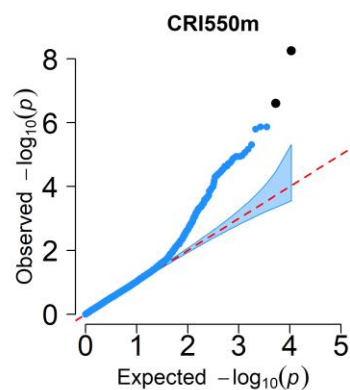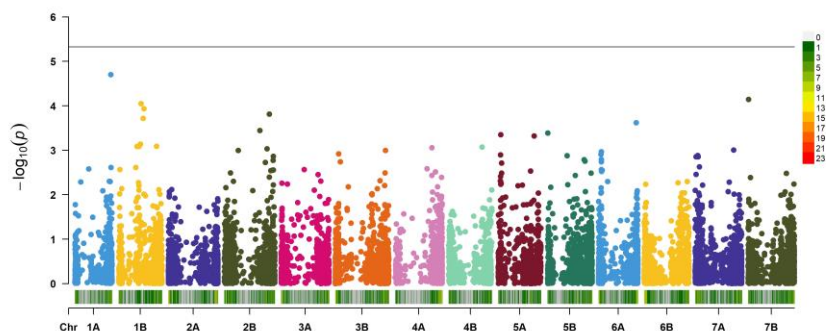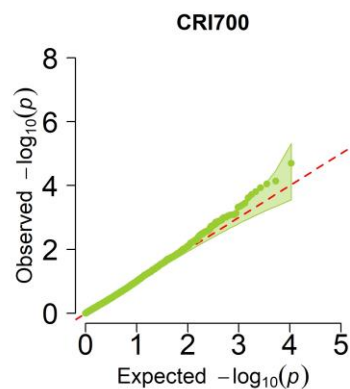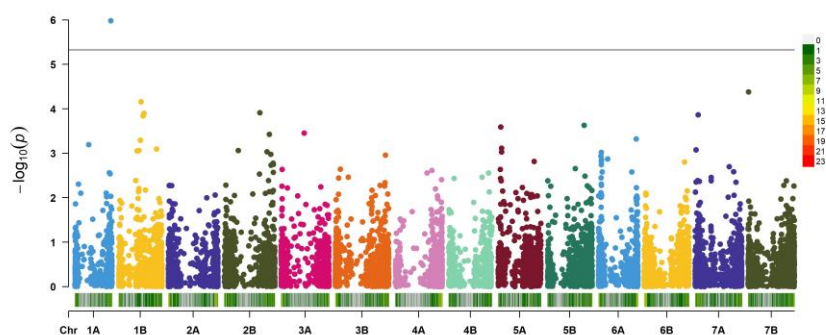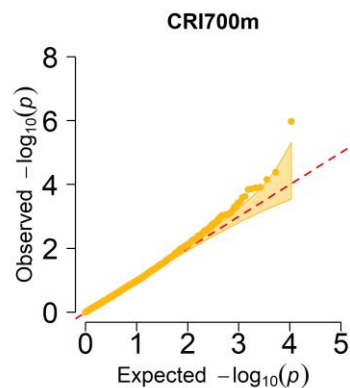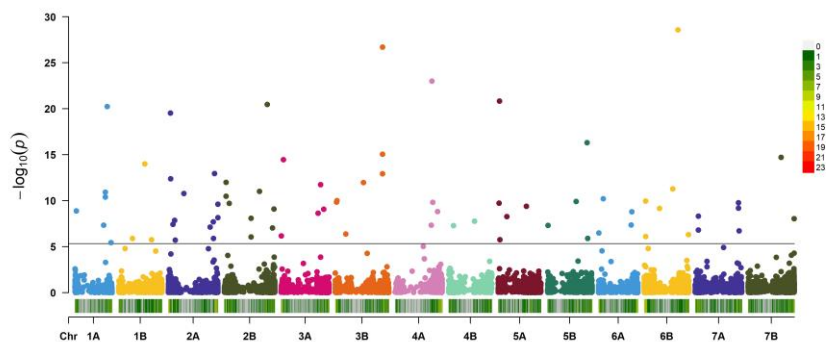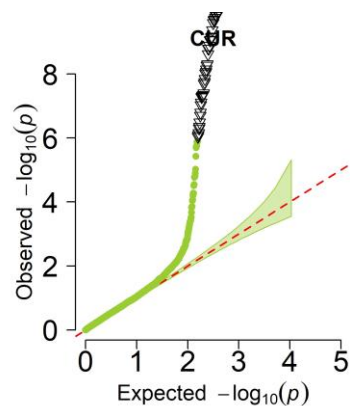

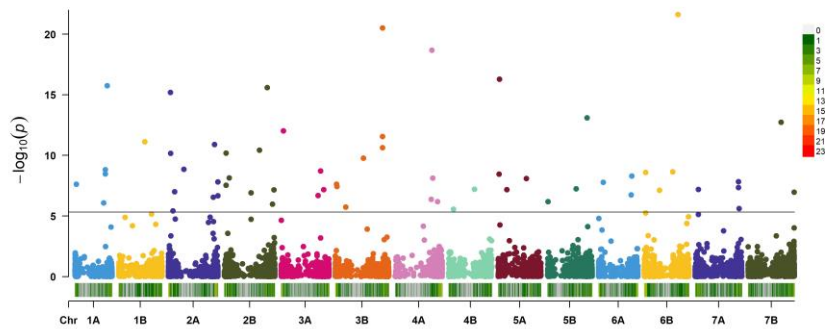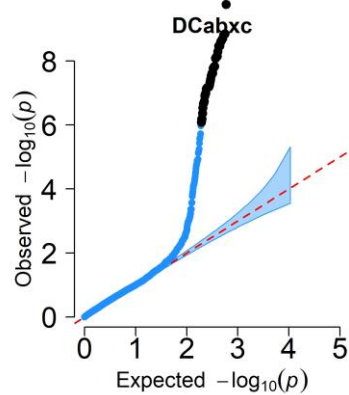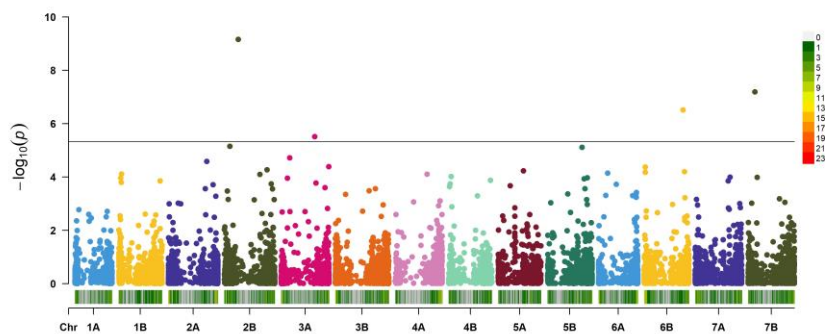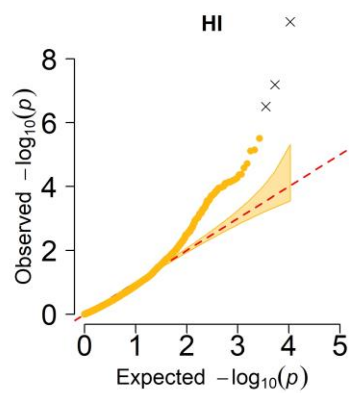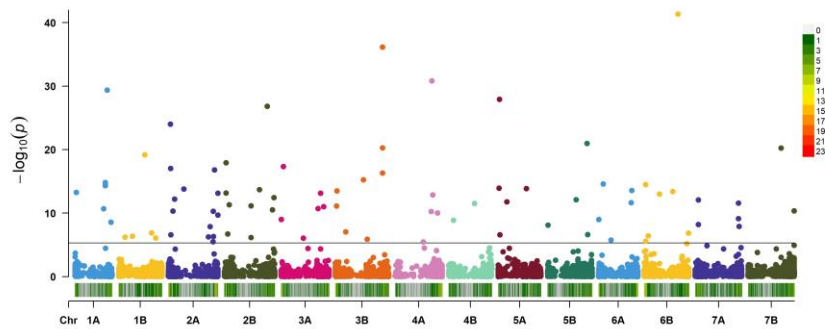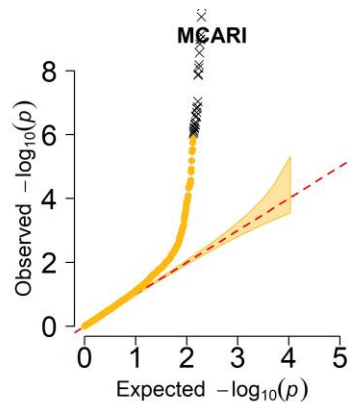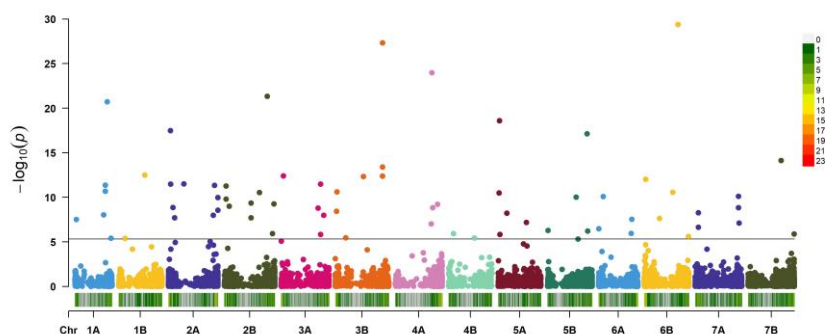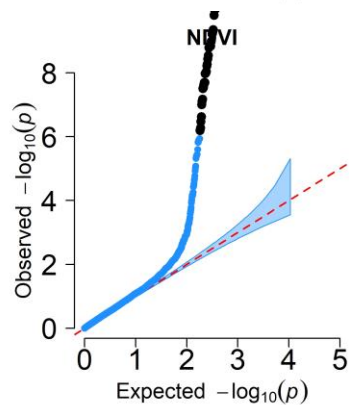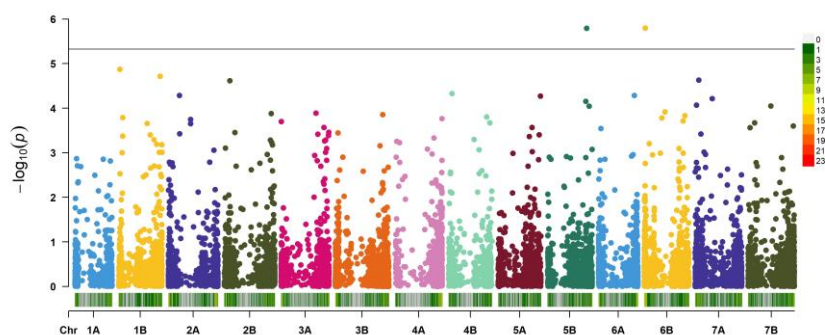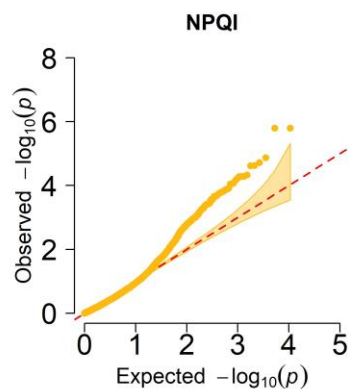

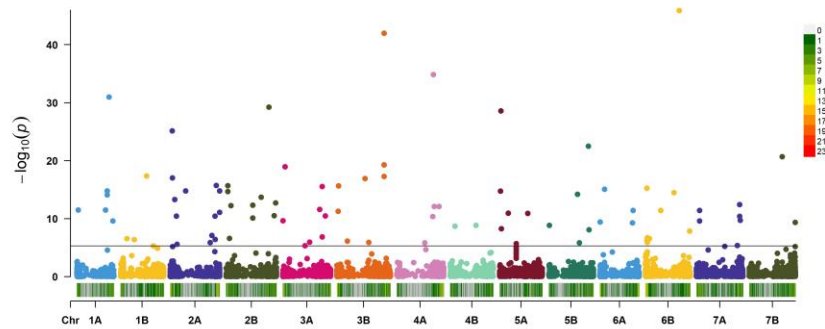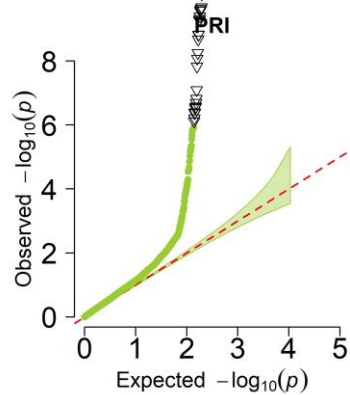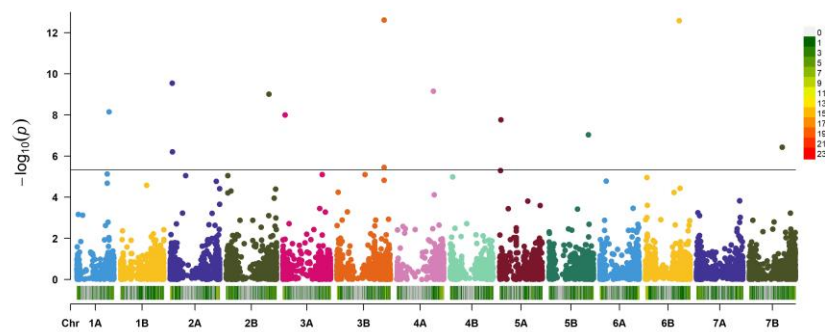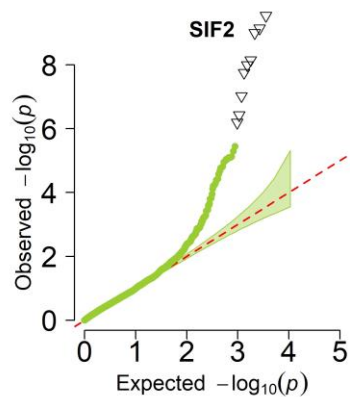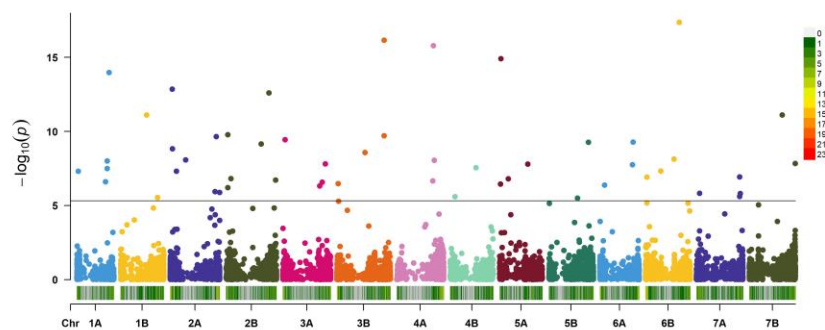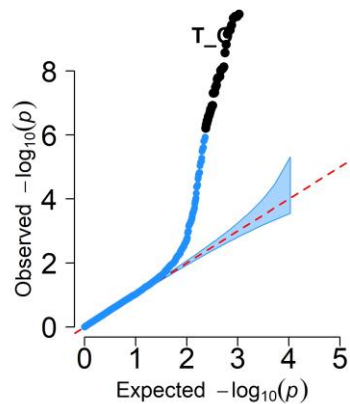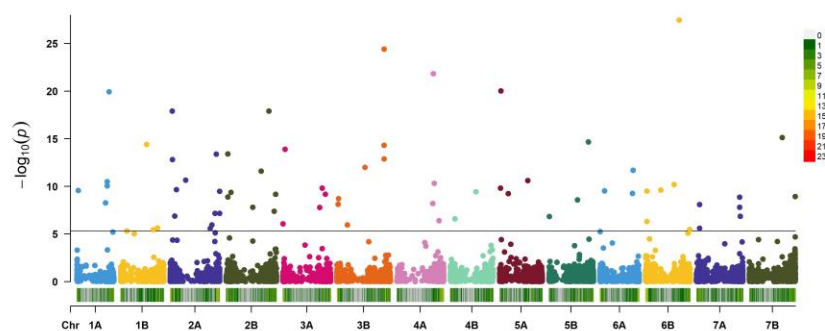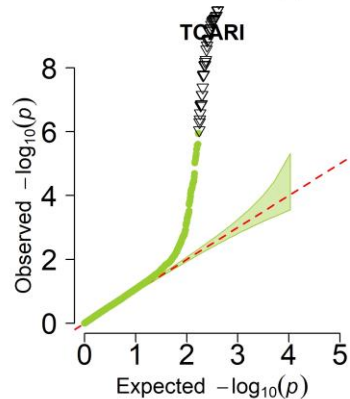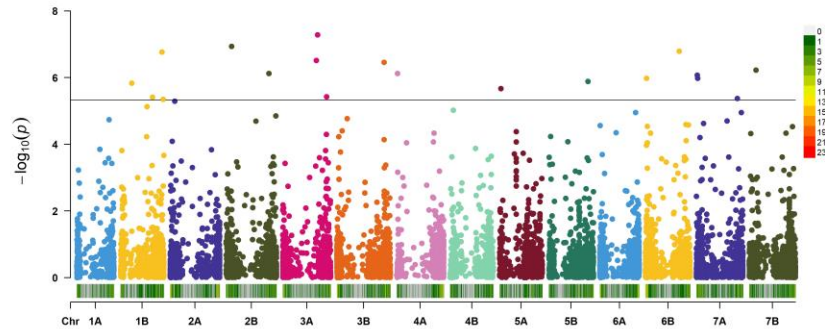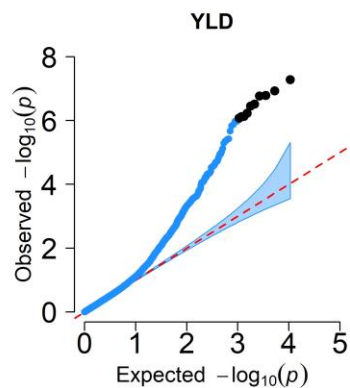

Supplement: Supplementary Figure S2 — Manhattan and quantile-quantile plots for the 19 traits assessed in GWAS analysis. [file DataSheet2.pdf]

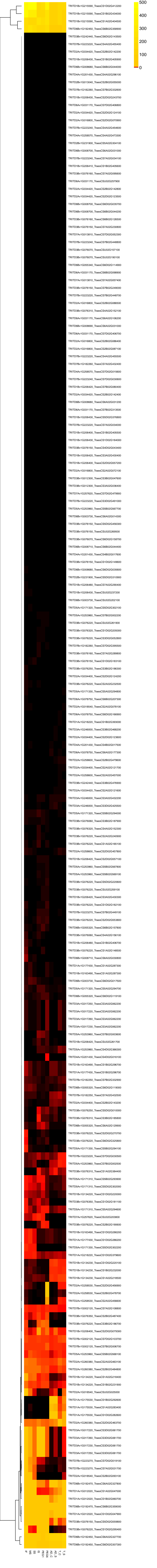

Supplement: Supplementary Figure S3 — Heatmap for gene expression analysis under several stress conditions for candidate genes. IF: irrigated field conditions; MS: mild stress conditions; SS: severe stress conditions (Gálvez et al., 2019 *); IS: seedling PEG shock control; PEG1: seedling 1 h PEG stress; PEG6: seedling 6 h PEG stress (Liu et al., 2015 **); AD_S: anther stage irrigated shelter phenotype; AD_S: anther stage drought stressed shelter phenotype; T_C: tetra stage irrigated shelter phenotype; and T_S: tetrad stage drought shelter phenotype (Ma et al., 2017 ***). [file DataSheet3.pdf]

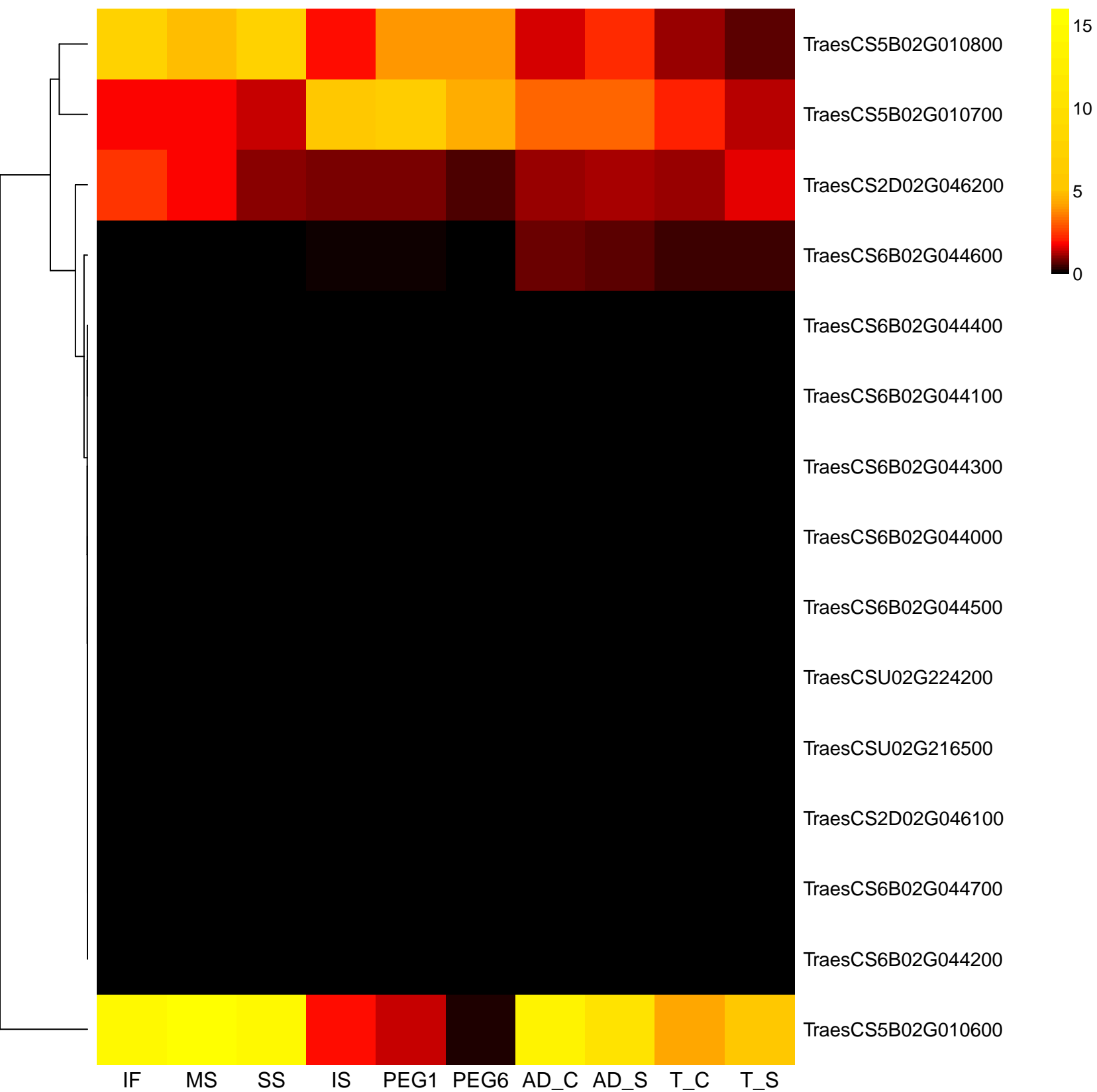

Supplement: Supplementary file 4 [file Image4.pdf]
